# The role of the estimand framework in the analysis of patient-reported outcomes in single-arm trials: a case study in oncology

**DOI:** 10.1186/s12874-024-02408-x

**Published:** 2024-11-23

**Authors:** Doranne Thomassen, Satrajit Roychoudhury, Cecilie Delphin Amdal, Dries Reynders, Jammbe Z. Musoro, Willi Sauerbrei, Els Goetghebeur, Saskia le Cessie, SISAQOL-IMI Work Package 3, Doranne Thomassen, Satrajit Roychoudhury, Cecilie Delphin Amdal, Dries Reynders, Jammbe Z. Musoro, Willi Sauerbrei, Els Goetghebeur, Saskia le Cessie, Rajesh Kamalakar, Kavita Sail, Ethan Basch, Jaap Reijneveld, Karen Keating, Yun Su, Ahu Alanya, Gerhard Rumpold, Maxime Sasseville, Jennifer Black, Geert Molenberghs, Khadija Rantell, Michael Schlichting, Antoine Regnault, David Ness, Silene ten Seldam, Tove Ragna Reksten, Anja Schiel, Ragnhild Sorum Falk, Alicyn Campbell, Joseph C. Cappelleri, Alexander Russell-Smith, Melanie Calvert, Samantha Cruz Rivera, Olalekan Lee Aiyegbusi, Limin Liu, Kelly Van Lancker, Claudia Rutherford, Vishal Bhatnagar, Ting-Yu Chen, Mallorie Fiero, Paul Kluetz

**Affiliations:** 1 Department of Biomedical Data Sciences, Leiden University Medical Center, P.O. box 9600, Postzone S-05-S, Leiden, 2300 RC The Netherlands; 2 Pfizer Inc, New York, NY USA; 3 Research Support Services, Oslo University Hospital, Oslo, Norway; 4 Department of Oncology, Oslo University Hospital, Oslo, Norway; 5 Department of Applied Mathematics, Computer Science and Statistics, Ghent University, Ghent, Belgium; 6 European Organisation for Research and Treatment of Cancer (EORTC) Headquarters, Brussels, Belgium; 7 Institute of Medical Biometry and Statistics, Faculty of Medicine and Medical Center, University of Freiburg, Freiburg, Germany; 8 Department of Clinical Epidemiology, Leiden University Medical Center, Leiden, The Netherlands

**Keywords:** Patient-reported outcomes, Single-arm trial, Estimand, Quality of life, Oncology, Repeated measurements

## Abstract

**Background:**

Patient-reported outcomes (PROs) play an increasing role in the evaluation of oncology treatments. At the same time, single-arm trials are commonly included in regulatory approval submissions. Because of the high risk of biases, results from single-arm trials require careful interpretation. This benefits from a clearly defined *estimand*, or target of estimation. In this case study, we demonstrated how the ICH E9 (R1) estimand framework can be implemented in SATs with PRO endpoints.

**Methods:**

For the global quality of life outcome in a real single-arm lung cancer trial, a range of possible estimands was defined. We focused on the choice of the variable of interest and strategies to deal with intercurrent events (death, treatment discontinuation and disease progression). Statistical methods were described for each estimand and the corresponding results on the trial data were shown.

**Results:**

Each intercurrent event handling strategy resulted in its own estimated mean global quality of life over time, with a specific interpretation, suitable for a corresponding clinical research aim. In the setting of this case study, a ‘while alive’ strategy for death and a ‘treatment policy’ strategy for non-terminal intercurrent events were deemed aligned with a descriptive research aim to inform clinicians and patients about expected quality of life after the start of treatment.

**Conclusions:**

The results show that decisions made in the estimand framework are not trivial. Trial results and their interpretation strongly depend on the chosen estimand. The estimand framework provides a structure to match a research question with a clear target of estimation, supporting specific clinical decisions. Adherence to this framework can help improve the quality of data collection, analysis and reporting of PROs in SATs, impacting decision making in clinical practice.

**Supplementary Information:**

The online version contains supplementary material available at 10.1186/s12874-024-02408-x.

## Background

Patient-reported outcomes (PROs) play an increasingly important role in the evaluation of treatments [1]. In the assessment of anti-cancer therapies, PROs are particularly relevant since the therapies are often aimed at prolonged survival and an improvement in quality of life (QoL) and/or symptom reduction. Regulatory authorities such as the US Food and Drug Administration (FDA) and the European Medicines Agency (EMA) have published guidelines on how to incorporate PROs in studies submitted for cancer therapy approval [2–9]. Guidelines for the inclusion of PROs in trial protocols and scientific reporting guidelines for PROs in clinical trials have also been developed [10, 11].

Results from single-arm trials (SATs) are becoming more prominent in the regulatory approval for oncology medicines, especially in rare cancer types for which there is not yet an effective standard of care, late-stage cancer, and for targeted therapies suitable only to a subgroup of patients with a specific mutation. One-third of the trials involved in FDA approval of oncological therapies between 2014 and 2019 were single-arm studies [12]. Although in some situations a carefully designed SAT is the most ethical or feasible option, results from SATs require careful interpretation because of the high risk of biases and the absence of concurrent control [13, 14]. This is a particular concern for PRO endpoints where dropout may be associated with the outcome measured.

Careful interpretation of an estimate calls for a clearly defined target of estimation, also called an “estimand” by the International Council for Harmonization (ICH). The ICH have set out their “estimand framework” in an addendum (R1) to their guideline E9 on Statistical Considerations for Clinical Trials [15]. The framework aligns the design and analysis of a trial with its aims. Fiero et al. have shown how the estimand framework may be used to translate a PRO-related research question into a fully defined estimand in a hypothetical randomized trial [16].

In a recent literature review on SATs in oncology that reported PROs [17], only two of the included studies specified a research hypothesis for the PROs. Intercurrent events in relation to the analysis of PROs were not discussed in any of the studies. The collection of PROs often stopped after treatment discontinuation, limiting the possibilities of a ‘treatment policy’ [15] or intention-to-treat analysis. Linear mixed models or other (implicit) imputation methods were used in many studies without acknowledging how the implicit imputation affected the interpretation of the results [17]. The intercurrent event of death is of particular concern here since PROs after death do not exist and implicit imputation after death is counterintuitive.

This case study was undertaken within the work package on SATs of the SISAQOL-IMI consortium, which aims to develop recommendations on design, analysis, presentation, and interpretation for PRO data in cancer clinical trials [18]. Our objective was to demonstrate how the estimand framework can be implemented in SATs with PRO endpoints. Specifically, we focused on global health-related QoL measured in a SAT in non-small cell lung cancer [19]. In this paper, we present a range of possible choices of estimand, corresponding statistical methods, and their implications retrospectively using anonymized data from a real single-arm cancer trial.

## Methods

In this section, we first briefly describe the design of the clinical trial used for illustration in this case study, in particular regarding PRO data collection. After touching on how missing PRO data were handled, we discuss the various estimands that were illustrated in this case study and the corresponding statistical methods for estimation. All statistical analyses were performed using R [20] and analysis code is available as an online supplement.

### Trial design

The single-arm, multicenter phase 2 trial evaluated the efficacy, safety, and tolerability of a new anticancer treatment in patients with locally advanced or metastatic anaplastic lymphoma kinase (ALK)-positive non-small cell lung cancer. The co-primary outcomes of the trial were objective tumor response (as per RECIST V1.1) and adverse events (using the National Cancer Institute Common Terminology Criteria for Adverse Events, V.4.0).

In addition, several PROs were assessed as secondary endpoints, of which we focused on the overall QoL as measured by EORTC QLQ-C30 global QoL scale. We will refer to this PRO as “global QoL.” The global QoL scale of the EORTC QLQ-C30 was scored according to the EORTC scoring manual [21] such that scores ranged from 0 (representing the worst) to 100 (best possible score). In the original study, a clinically relevant difference of 10 points in mean QoL compared to the mean at the start of protocol treatment was defined [19]. The trial was conducted before the estimand framework was developed, and no strategy to deal with intercurrent events or missing PRO data was explicitly mentioned in the paper.

### Collection of PRO data in the trial

While on trial medication, participants were asked to complete the EORTC QLQ-C30 questionnaire on the first day of protocol treatment and every three weeks while on protocol treatment. The tri-weekly intervals were aligned with the treatment cycles of chemotherapy, which was the standard of care for this disease setting. We therefore refer to the timing of PRO measurements with their cycle number and baseline is defined here as the first day of cycle 1. After cycle 10 (30 weeks), the study protocol allowed for completion of the questionnaire on the first day of alternate cycles (i.e., every six weeks).

With respect to PRO data collection, three types of intercurrent events occurred in the study: progression of disease (PD), treatment discontinuation (TD, mostly due to disease progression), and death. Although disease progression was often followed by the discontinuation of treatment in the trial, it was left to the discretion of the physician and patient to decide when to stop the treatment. Upon discontinuation of trial medication, patients were asked to complete one final questionnaire, after which PRO data collection was ended, while only follow-up for overall survival continued. The sponsor anonymized the measurements before a subset was shared with us for this case study.

### Methods for missing PRO data

#### Description of missing data

For each cycle, we summarized the number of patients in each of the following six states: 1. alive, on treatment and QoL available; 2. alive, on treatment and QoL not available; 3. alive, off treatment and QoL available (this was extremely rare); 4. alive, off treatment and QoL not available; 5. lost to follow-up for overall survival (and QoL); 6. deceased. In the rare case when there was more than one PRO measurement reported by the same patient in one cycle, we averaged the patient’s PRO measurements in that cycle*.* As intercurrent events play an important role in the definition of an estimand, the availability of PROs before and after intercurrent events was also analyzed descriptively.

#### Imputation of missing data

In line with our illustrative aim, we created one (reasonably realistic) complete dataset in which the implementation of the estimand framework could be studied. To this end, we applied a single imputation method (Appendix A.1). We observed a general drop in PRO scores in the last five cycles before death, whereas no such drop was observed before censoring. The progression of disease and the decision to discontinue treatment may be related to patients’ QoL trajectories as well. We therefore assumed that the time-distance to death and other intercurrent events was relevant to the missing PROs at each cycle.

We imputed missing PRO data using single imputation (see Appendix A.1 for details) for each participant until cycle 40 or death, whichever occurred first, under the assumption that the PROs at each cycle were missing at random conditional on the cycle number, available QoL measurements at other cycles, death, PD and TD, and the time until these events. Missing values were imputed before and after PD and/or TD, but not after death. We assumed non-informative censoring in our analyses, as most censoring was administrative at study end.

### Applying the estimand framework in our case study

We introduced various estimands (i.e., targets of estimation) for describing global QoL over time from the start of protocol treatment. As defined in ICH E9-R1, an estimand has five attributes: the treatment, the population, the variable of interest, the population-level summary, and a strategy for handling intercurrent events [15]. In this case study, the assigned treatment was the same trial medication for all participants, and we used the in- and exclusion criteria of the original trial to define our target population of ALK positive non-small cell lung cancer patients. Appendix A.2 provides a general discussion on defining the variable, the population summary and the handling of intercurrent events for PROs in a SAT. Below, we outline the estimands that were illustrated in this case study specifically, as well as corresponding statistical methods for estimation (for a schematic overview see also Table 1).

**Table 1:** Combinations of intercurrent event strategies that were illustrated in this case study and their corresponding analysis methods

Figure	Intercurrent event strategies				Analysis	
*Strategy*	*While no IE*	*Composite*	*Hypothetical*	*Treatment policy*	*QoL data included in the analysis*^*a*^	*Estimation of mean QoL at each cycle*
Figure 3						
* 1a,b*	Death			PD, TD	All outcomes until each patient’s respective death in the first 40 cycles^b^, including outcomes after TD or PD	1a. GEE with independence correlation structure, where cycle number was included as a categorical variable [26] 1b. An LMM with random intercept and slope was estimated, where the cycle number was included as a categorical variable. Individual outcome predictions from this LMM were computed at each cycle and averaged only over those patients alive at that cycle.
* 2*		Death		PD, TD	All outcomes until each patient’s respective death in the first 40 cycles, including outcomes after TD or PD. After death, the outcome was set to 0 until cycle 40	Estimated means using a GEE with independence correlation structure, where cycle number was included as a categorical variable (Applying an LMM would also be possible here)
* 3a,b*			Death	PD, TD	All outcomes until each patient’s respective death in the first 40 cycles, including outcomes after TD or PD	3a. Marginal means from an estimated LMM with random intercept, where the cycle number was included as a categorical variable 3b. same as 3a, but with additional random slope
Figure 4						
* 1*	Death, TD			PD	All outcomes before each patient’s respective TD or death in the first 40 cycles, including outcomes after PD	GEE with independence correlation structure, where cycle number was included as a categorical variable. (Averaging individual LMM predictions over those still on treatment at each cycle would also be possible)
* 2a*			Death, TD	PD	All outcomes before each patient’s respective TD or death in the first 40 cycles, including outcomes after PD	Marginal means from an estimated LMM with random intercept, where the cycle number was included as a categorical variable
* 2b*	Death		TD	PD	All outcomes before each patient’s respective TD or death in the first 40 cycles, including outcomes after PD	Estimating an LMM and averaging individual predictions at each cycle over those still alive at that cycle
* 3*	Death			PD, TD	*Same as estimates 1a,b in *Fig. 3	
Figure 5					*The analyses for *Fig. 5* below are analogous to those for *Fig. 4* but with “TD” replaced by “PD” and *vice versa	
* 1*	Death, PD			TD	All outcomes before each patient’s respective PD or death in the first 40 cycles, including outcomes after TD	GEE with independence correlation structure, where cycle number was included as a categorical variable. (Averaging individual LMM predictions over those still without PD and alive at each cycle would also be possible)
* 2a*			Death, PD	TD	All outcomes before each patient’s respective PD or death in the first 40 cycles, including outcomes after TD	Marginal means from an estimated LMM with random intercept, where the cycle number was included as a categorical variable
* 2b*	Death		PD	TD	All outcomes before each patient’s respective PD or death in the first 40 cycles, including outcomes after TD	Estimating an LMM and averaging individual predictions at each cycle over those still alive at that cycle
* 3*	Death			TD, PD	*Same as estimates 1a,b in *Fig. 3	

^a^Assuming complete QoL data for each patient from study registration until cycle 40 or death; to obtain such complete data(sets) for analysis in practice likely requires imputation to be performed before the analyses listed here

^b^Some patients in the study were censored for overall survival before cycle 40. As censoring was mostly administrative at the end of the study, we assumed uninformative censoring in our analyses. If an informative censoring mechanism is plausible, weighted GEE approaches or joint models may be used to account for such censoring [26, 27]. *PD* disease progression, *TD* treatment discontinuation, *GEE* generalized estimating equations, *LMM* linear mixed model

#### Defining the variable and the population summary

To illustrate the different variables of interest, we computed summaries of the numerical value of the PRO, the change from baseline, and a responder/non-responder classification at each cycle using the raw, unimputed data. We opted for the absolute numerical value of the PRO for subsequent analyses [22–25]. Because of the limited availability of the PRO data in later cycles, we restricted our analyses to cycles 1–40 (months 0–27). Furthermore, we opted for the mean QoL value at each cycle as the population summary. Since the distribution of observed QoL was reasonably symmetric in exploratory analyses, the mean was deemed to be an appropriate summary. For a range of intercurrent event strategies, we applied a corresponding analysis (outlined below) to show how results and interpretations differ.

#### Strategies to deal with death

In our illustrations of strategies to handle death in the analyses, we used all (imputed) data after TD and PD, regardless of whether TD and PD had occurred, following a treatment policy strategy. For transparency, we provided survival estimates with our global QoL estimates, as well as estimates of the probability of remaining progression-free and of remaining on protocol treatment.

### While alive strategy

First, for each cycle, we estimated the mean QoL in the patients who were still alive in that cycle. Note that over time the group in which the means are calculated becomes smaller due to mortality and censoring, and may have a different distribution of characteristics than the group who is alive at the first cycle. Therefore, we provided (Kaplan–Meier) estimates of survival with the estimated means while alive. Essentially, we are interested in a bivariate outcome here: survival and QoL conditional on survival.

The while alive estimates were obtained in two ways: (1) using generalized estimating equations (GEE) with an independence correlation structure, which have been shown to allow direct modelling of means over time conditional on survival status [26, 27], and (2) modelling the individual QoL values over time with a linear mixed model (LMM) followed by averaging individually predicted QoL values only over those alive at each cycle [27]. A GEE analysis with an independence correlation structure, with time as a categorical variable and without any approaches to handle missing data will yield the same means over time as a purely descriptive approach. So, this GEE approach is in line with a descriptive aim. In addition, the GEE approach models the means over time only, whereas the LMM provides individual predictions that we averaged over the alive subject afterwards (since using marginal means from the model directly would correspond to a hypothetical strategy, see below).

### Composite strategy

As an example of a composite strategy for death, all global QoL values after death were set to 0, a value chosen somewhat arbitrarily for the sake of illustration here (cf. EQ-5D, a health utility score where a value of 0 corresponds to death [28]). The means of this composite outcome were estimated using GEE as above. Whether it makes sense to put global QoL and death on the same scale, and to assign a value of 0 to death on this scale, is debatable.

Different choices of the QoL variable of interest allow for other ways to define a composite endpoint of QoL and death. For example, if a responder analysis is performed, death might be included in the definition of nonresponse. In an analysis of time-till-deterioration, deterioration may be defined as a drop in QoL or death. However, such analyses have their limitations as discussed in Appendix A.2 [22–25].

### Hypothetical strategy

Finally, we estimated the mean QoL over time in a hypothetical situation where all patients would remain alive until at least cycle 40. Two LMMs including the cycle number as a categorical variable and 1) a random intercept only, or 2) a random intercept and slope, and were fitted to the available data. Subsequently, marginal means for cycles 1 through 40 were obtained from the fitted models. These models assume that the QoL values of patients who are no longer alive at a particular cycle are missing at random conditional on observed QoL values and the cycle number. Under this assumption, LMMs (implicitly) extrapolate QoL trajectories for each patient after their death to model a hypothetical scenario assuming no deaths in the trial.

#### Strategies to deal with treatment discontinuation

Various strategies to handle TD in PRO data analysis were applied along with strategies to handle death described above. Progression of disease was ignored here using a treatment policy strategy for PD. We did not define a numerical composite outcome for TD, as associating a single global QoL score with TD did not seem reasonable.

### While on treatment

First, we implemented a while on treatment strategy, which implies a while alive strategy since treatment does not continue after death. The mean QoL at each cycle (while on treatment) was estimated by removing all observations after treatment discontinuation from the data and fitting a GEE with independence correlation structure to the remaining data.

### Hypothetical strategies

Additionally, two hypothetical strategies were illustrated. For both strategies, an LMM was fitted to all data before TD. For the first strategy, the model’s predictions were averaged over all patients at cycles 1–40. This resulted in estimated mean QoL in the hypothetical situation assuming no treatment discontinuation or death in the study. For the second strategy, we averaged the same model’s predictions over the subset of patients who were still alive at each respective cycle. This was intended to estimate the mean QoL while alive, in the hypothetical situation where treatment discontinuation did not occur before death within the study.

### Treatment policy strategy

Finally, we applied a treatment policy strategy to TD, while alive. Here, we used the data with (imputed) measurements after TD and applied GEE to estimate the mean QoL while alive, regardless of TD. This is the same analysis as for the while alive method described previously. As almost no data were available after TD, these estimates were mostly based on imputed data at later cycles.

#### Strategies to deal with disease progression

Subsequently, we set the strategy for TD to a treatment policy strategy and shifted our focus to the intercurrent event of disease progression (PD). We defined intercurrent event strategies for PD analogously to those for TD.

## Results

### Inclusion and follow-up

A total of 876 patients from the lung cancer SAT with at least one QoL or clinical measurement were included in our analysis. This excludes patients from participating centers not allowing the use of data for this purpose.

The median [IQR] follow-up time in those censored for overall survival was 41.8 [28.1–47.3] months. Most censoring (72%) was near the data collection cut-off date. We therefore assume that most censoring (for overall survival) was administrative censoring and uninformative censoring was likely to hold. In a multivariable Cox regression, censoring was not associated with sex, age, baseline ECOG performance status or the number of previous therapies.

### Description of clinical characteristics

Demographic and clinical characteristics of the study participants in our data were summarized (Appendix A.3 Table S1). Death was observed in 576 (66%) patients. The Kaplan–Meier estimate of median survival time was 21.7 months [95% CI 19.8–24.2], with the probability of survival of 67% [95% CI 64%-71%] and 47% [95% CI 43%-50%] after one and two years of the start of protocol treatment (A.3 Figure S2).

### Description of global quality of life

At baseline, 834 (95%) of the patients filled in the QoL questionnaire. The mean (SD) global QoL was 53.7 (25.2) at cycle 1. A positive association between QoL at baseline and overall survival was observed (A.3 Figure S3, p < 0.001 for log-rank test where participants were stratified based on four equal-length intervals of baseline QoL).

### Description and imputation of missing PRO data

#### Availability of PRO data

**Fig. 1:**
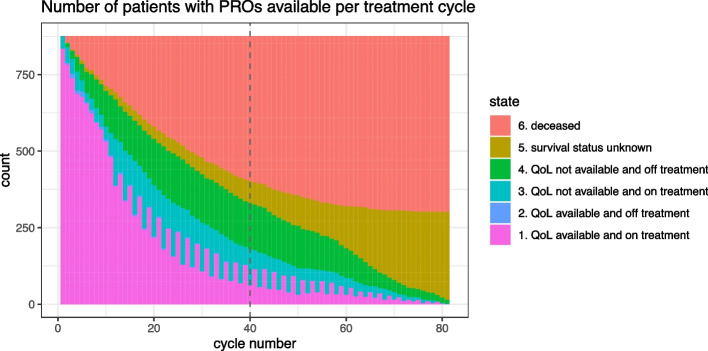
Availability of PROs over time, in relation to treatment and survival status. Note that by design of the study, almost no PROs were reported after discontinuation of protocol treatment. As a result, there are no to very few patients in state 2 at each cycle

The number of available PROs reduced to 538 (61%) by cycle 10 and 221 (25%) by cycle 20. In cycle 40, when the estimated survival probability was 43%, only 61 patients (7%) were still alive and completed the QoL questionnaire (Fig. 1). For this reason, we restricted our further analyses to cycles 1–40.

The median time between the last available PRO and death observed during follow-up was 2.7 months (A.3 Figure S4). PRO data was available until less than 1 month before death for 140 patients (24% of observed deaths), and less than 3 months before death for 315 patients (55%). PRO measurements were mostly available until shortly before censoring of the survival time: the median [IQR] time between the last available PRO and censoring was 1.59 [0.62–16.24] months.

PROs were collected until PD for most patients in whom PD was recorded (*n* = 648). After PD, 345 patients continued the trial medication for at least one month and 241 continued for 3 months or more. Most (89%) patients reported a PRO measurement at the discontinuation of protocol treatment. Eight patients had two PRO measurements post-discontinuation, and one patient had three PRO-measurements after they discontinued treatment.

#### Imputation

After imputation, the mean PRO in those alive at each cycle was slightly lower than in the available data (A.1, Figure S1). Intercurrent events such as PD and (being close to) death may lead to missing PROs and be associated with lower QoL. As our imputation model takes such events into account, we would indeed expect global QoL in the imputed dataset to be somewhat lower on average than in the available data, particularly at later cycles when more participants have experienced PD and/or are in the final weeks of their life.

### Illustration of the estimand framework

Below we show results corresponding to the estimands defined in Sect. " Applying the estimand framework in our case study". Our focus is on the illustration of different variables of interest and on strategies to deal with intercurrent events, in particular death, TD and PD.

#### Defining the variable of interest: illustration

**Fig. 2:**
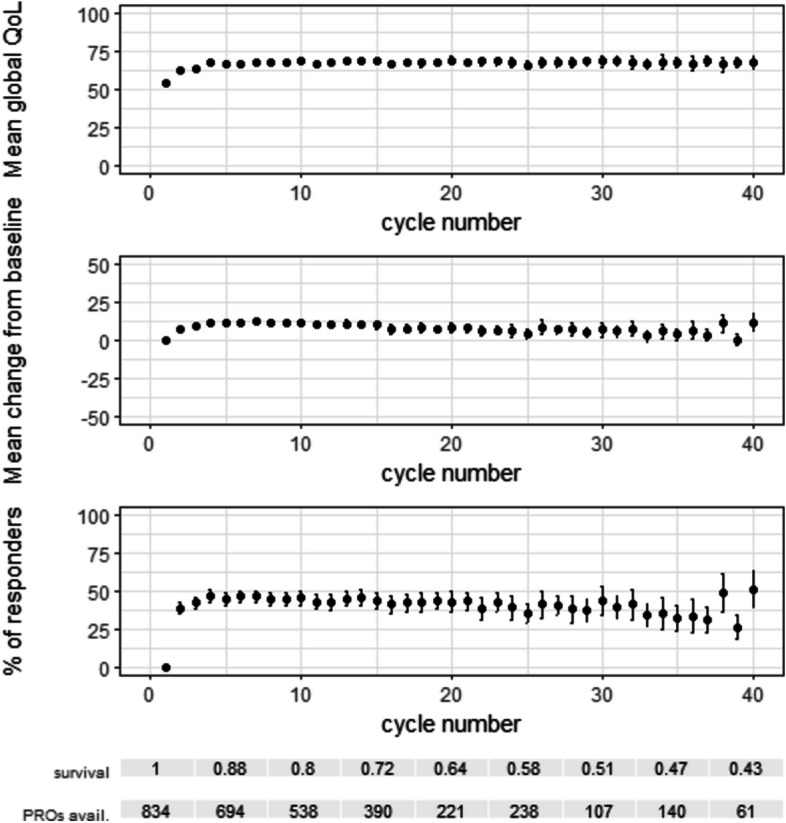
Mean and 95% CI of three possible variables of interest, within available (unimputed) data at each cycle. Top graph: absolute global QoL. Middle graph: difference in global QoL from the start of protocol treatment. Bottom graph: responder classification, where response for a patient is defined as an increase of at least 10 points in global QoL from the start of protocol treatment

Our variable of interest was the absolute numerical value of the PRO. For illustration, we provide summaries of three possible variables of interest in our raw data: the absolute numerical value, the magnitude of change from baseline and a binary responder/non-responder classification at each cycle (Fig. 2).

#### The absolute (numerical or ordinal) values of the PRO

The mean reported global QoL at the beginning was 54 (*n* = 834), increased to 67 (*n* = 654) in cycle 4 and then appeared relatively constant until cycle 40. Note that the population with available measurements shrinks over time from death and drop-out, mainly due to TD and PD.

#### Magnitude of change from baseline

A downward trend in the mean change from baseline was visible after cycle 6 within the available data. These results illustrate that when the mean QoL in those alive remains constant, and there is a selection process over time where patients with high starting values live longer, the mean change from baseline in those alive will decrease over time. Regarding floor and ceiling effects, 33 patients (4.0% of available baseline measurements) were at the lowest possible QoL level at baseline, whereas 43 patients (5.2%) had the maximum possible global QoL score at baseline. The patients with the minimum possible QoL at baseline cannot have a negative change by definition. At the same time, patients at the top of the global QoL scale at the start can never have a positive change. This is important to consider when interpreting the mean change from baseline.

#### Responder/non-responder classification

As an example, we defined response at each cycle as having an increase in at least 10 points in QoL compared to baseline. A responder definition based on the magnitude of change from baseline may also suffer from ceiling effects: in our example, participants with baseline QoL values of 91 or higher could never be classified as a responder. The proportion of responders at each cycle within those patients who reported PROs showed an initial increase in the first four cycles and then a gradual downward trend.

#### Strategies for dealing with intercurrent events

For the intercurrent event strategies defined in Sect. " Applying the estimand framework in our case study", we estimated the corresponding mean global QoL at each cycle within the imputed dataset. The various intercurrent event strategies resulted in diverging estimates, each with their own interpretation.

#### Death

**Fig. 3:**
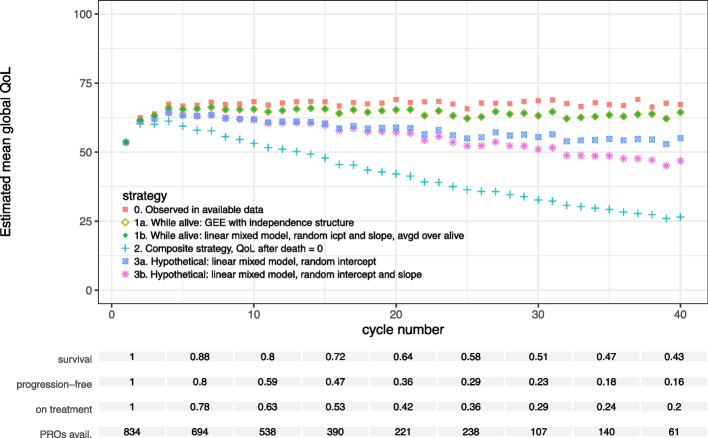
Estimated mean global quality of life in each cycle for different estimands and corresponding models. The estimands differ in the chosen strategy to deal with the event of death in the analysis of the PROs. The two statistical methods applied to estimate mean QoL while alive (1a and 1b) overlap in the figure, as their resulting estimates were very similar

The estimated mean QoL while alive increased after treatment initiation and showed a slight decreasing trend at later cycles (Fig. 3). The GEE and the LMM with post-hoc averaging yielded very similar results in this case. The composite estimates decrease rapidly after cycle 4. This is due to death dominating the composite outcome with an increasing proportion of patients with a QoL of 0 in the dataset.

Both hypothetical strategies resulted in estimated mean QoL values below the while alive estimates. During the hypothetically extended part of the participants’ lives, their average QoL at each cycle was estimated to be lower than the average QoL of participants who were alive in the same cycle. The model with the random intercept and slope resulted in lower mean QoL estimates than the model with a random intercept only. The (linear) random slope model fitted the data better (difference in AIC: 1510, p-value for likelihood ratio test: < 0.0001). Models with random effects for flexible spline functions of the cycle number were unstable. Hence we could not test for nonlinear effects. Both models aim for the same hypothetical estimand for death and both models extrapolate QoL after death. Yet *how* the models extrapolate is determined by the model specification. No major differences in CI width occurred between the various analysis methods used.

Regarding statistical efficiency, it is difficult to compare estimation methods that correspond to diverging estimands, as these methods are not targeting the same quantity. However, we observed no major differences in standard error magnitude between the analysis methods illustrated in this subsection (A.3 Figure S5).

#### Treatment discontinuation

**Fig. 4:**
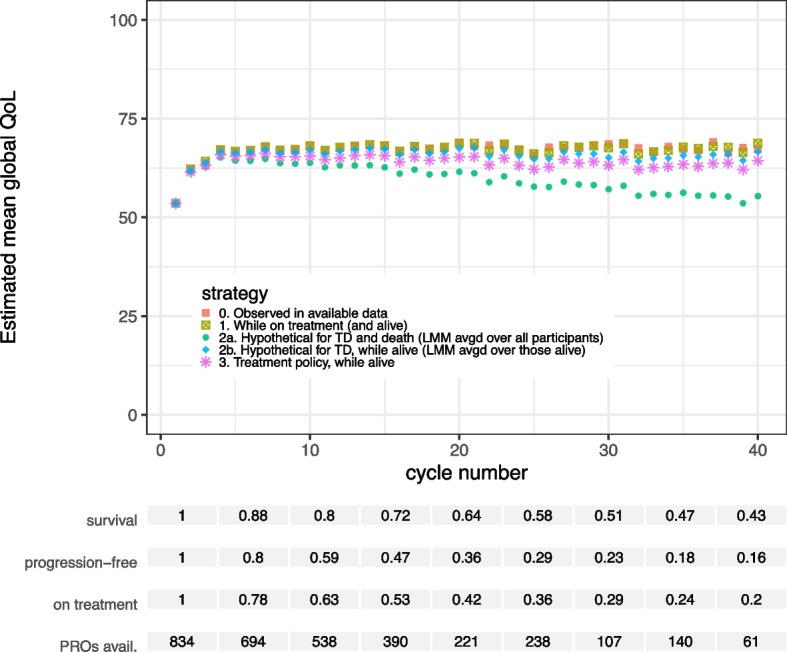
Estimated mean global quality of life in each cycle for different estimands and corresponding models. The estimands differ in the chosen strategy to deal with the event of treatment discontinuation (TD) and death in the analysis of the PROs

The estimated mean QoL while on treatment at each cycle was slightly higher than the while alive estimates ignoring treatment discontinuation (Fig. 4). This reflects that QoL likely decreases to some extent after TD, as drivers of TD may also lead to decreasing QoL. The estimated means in a hypothetical situation without treatment discontinuation, while alive were higher than the means while alive (treatment policy). The estimated mean global QoL under a hypothetical strategy for both TD and death is lower than the while alive estimate for each cycle. Confidence interval widths were similar, although the estimates for the treatment policy (while alive) strategy were somewhat more precise than the others (A.3 Figure S6). This could be due to the treatment policy estimates using all (imputed) data up to death, whereas the other estimates are based on data from before TD only.

#### Disease progression

**Fig. 5:**
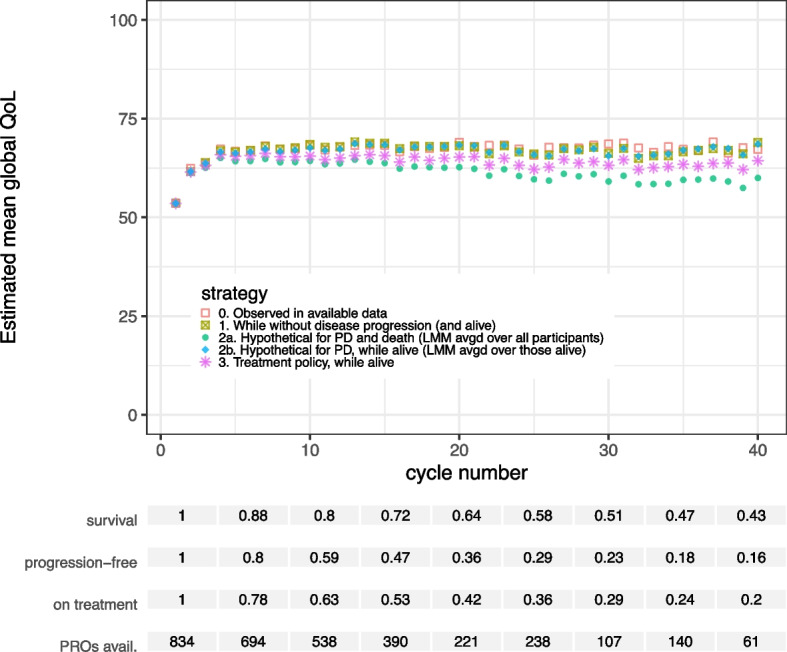
Estimated mean global quality of life in each cycle for different estimands and corresponding models. The estimands differ in the chosen strategy to deal with the event of disease progression (PD) and death in the analysis of the PROs

Comparing the resulting estimates for disease progression (Fig. 5) to those for treatment discontinuation (Fig. 4), we note that QoL in the hypothetical world where PD does not occur in the trial is estimated to be higher than in the hypothetical world where TD does not occur. PD usually occurs earlier than TD in our dataset. Any drop in QoL after PD but before TD that is observed in the data is not taken into account by the linear mixed model in the first hypothetical scenario, since it is only fitted on observed values before PD.

## Discussion

In this case study, we have outlined the meaning and impact of the estimand framework in the analysis of longitudinal PROs in a SAT. While the causal interpretation of SAT results remains challenging due to a high risk of biases, the estimand framework facilitates a clear definition of the aims and interpretation of a SAT analysis. Hence, the use of this framework mitigates some of the methodological issues previously observed for PROs in SATs, such as the lack of an explicit strategy to address intercurrent events [17].

The results of our illustration show that decisions made in the estimand framework are not trivial. In particular, each intercurrent event handling strategy resulted in its own estimated QoL means over time, with a specific interpretation, suitable for different clinical research aims.

### The absolute numerical value of the PRO as the variable of interest

The absolute numerical value of the PRO, change scores and responder classification as possible endpoints of interest were illustrated on our dataset. The interpretation of change scores or responder/non-responder classifications may be obscured by floor and ceiling effects, a selection process due to death on baseline values, regression to the mean and the non-definitive nature of changes in the PRO (see Appendix A.2 for more discussion). Generally, the absolute numerical value of the PRO suffers from fewer drawbacks than other options and is the most direct representation of the data [22–25], especially for a descriptive study aim. A corresponding population summary might be the mean PRO at (a) prespecified time point(s).

### The while alive strategy and the use of LMMs and GEE

When dealing with death in the analysis of PROs, a while alive strategy most closely represents the actual or observed situation. Any (implicit) imputation of PROs after death implies that these values are missing and observable in principle. However, PROs after death are neither observable nor defined. The assumption of a hypothetical world in which patients do not die during the study was far removed from reality in our case study. This hypothetical scenario may therefore not be clinically relevant, particularly in groups of patients where the mortality rate is high. Especially in a SAT context, where the aim is often descriptive, it makes sense to stay close to the experienced reality and use a while alive strategy combined with an estimate of the survival probability. In a randomized trial context, a drawback of the while alive strategy may be that differences in case-mix arise between the trial arms over time because of differential survival. However, this would also be the case in future implementations of the treatment in similar populations.

Crucially, marginal effects from a standard linear mixed model of PROs over time rely on implicitly imputed PRO values after death. It is important for researchers to be aware that the use of such a model implies a hypothetical estimand regarding death. For other intercurrent events, fitting an LMM that does not account for intercurrent events may correspond to different estimands depending on data availability. For example, if there are no data after TD, fitting an LMM corresponds to a hypothetical strategy for TD and death, whereas the same model may estimate a treatment policy estimand for TD if all data after TD are available until death. Population means while alive can be obtained by averaging individual predictions from a fitted LMM over those still alive. If direct estimates of population level means are of interest in a while alive strategy, we recommend the use of GEE with an independence correlation structure [26, 27]. The appropriate analysis will depend on the trial objective and stakeholders.

### The treatment policy strategy for non-terminal intercurrent events

For non-terminal intercurrent events such as treatment discontinuation, a treatment policy strategy seems a reasonable choice, as it aligns with the intention-to-treat principle. Often, it is relevant to know what to expect of a treatment, even after it is discontinued. Of course, this depends on the trial aim, for example, a while on treatment strategy may be appropriate for a tolerability objective. A treatment policy strategy for TD and PD requires data collection after these events, which is often limited as in our case study. After discontinuation of the trial treatment, patients may transfer to a different treatment center and/or enter another trial. This complicates the observation of PROs after TD, especially in single-arm trials and rare diseases. In this paper, we showed analyses where a ‘treatment policy’-strategy was always applied to TD or PD (or both). Of course, other combinations are possible, and the combination of strategies should be defined carefully.

### The composite strategy

A composite outcome of a PRO (or any other outcome) and an intercurrent event may be dominated by the intercurrent event [29, 30]. Examples of composite outcomes include the EQ-5D measure [28, 31] and Quality-Adjusted Life Years [32] or variants such as Quality-adjusted Time Without Symptoms or Toxicity [33]. For transparency, results from a composite strategy should be accompanied by a measure of the incidence of the event. Furthermore, the interpretation of a composite outcome is difficult when the intercurrent event cannot meaningfully be put on the same scale as the PRO. For instance, to assign a single value to QoL after someone’s treatment has been discontinued, makes little sense. In the composite strategies of this case study, the assumption of QoL at 0 after death (as is also done in the EQ-5D measure [28]) is highly debatable and makes no sense from a clinical point of view as patients do not experience QoL after death. While composite endpoints may be common in some contexts such as Health Technology Assessment, we suggest caution in assigning a single PRO value to death or other intercurrent events.

### Choosing the most appropriate estimand in a study

The advantages and limitations of each estimand discussed above can be considered when applying the estimand framework to PROs in a clinical study. The research setting determines the most relevant estimand, depending on, for example, the stakeholders involved, the type of PRO, and the PRO objective (e.g., to assess the efficacy or the tolerability of a new treatment). If PRO data are intended for review of payer or regulatory submission, discussions with these stakeholders to identify appropriate estimands and analyses are highly encouraged.

Some studies explore more than one estimand to address multiple stakeholders. In that case, we recommend clearly specifying the targeted estimand with each presented result, for instance, in tables and figure legends. Estimates of different estimands have different interpretations: they are not estimates of the same quantity. It is therefore important to clarify the intended interpretation of each result when reporting on a study.

### Intercurrent events and the imputation of missing PROs

Finally, we note that the choice of intercurrent event strategy and dealing with missing PRO data are two separate but related topics. Intercurrent events may hinder the measurement of PROs, causing missingness, and intercurrent events may be predictive for the missing values, e.g., QoL may decrease at a time of disease progression. Conditioning on information about intercurrent events in imputation may make a MAR assumption more plausible.

In our analyses, we assumed non-informative censoring, as most censoring was administrative at the end of the study. If an informative censoring mechanism is more plausible, weighted GEE approaches or joint models may be used to account for such censoring [26, 27].

The single imputation method that we applied to address missing data was meant to generate a single example dataset for illustration of the estimand framework. Multiple imputation would better account for uncertainty in the missing values. An alternative way to account for missing PRO data would be to reweight observations by the inverse probability of missingness in the analysis. In addition, our MAR assumption may not hold. Finally, there were virtually no data after treatment discontinuation, so the relation between TD and PROs after TD could not be estimated from the data. Patients’ health status might deteriorate after treatment stops, but they may also switch to a new treatment that improves their QoL. We plan to explore imputation methods for longitudinal PROs in the presence of intercurrent events in detail in a future study.

## Conclusions

This case study has illustrated possible estimand definitions when dealing with PROs in a single-arm cancer trial and discussed considerations underpinning this choice. We have also provided an overview of corresponding statistical methods for the estimation of each estimand. Our findings show that trial analysis results and their interpretation strongly depend on the chosen estimand. The estimand framework provides a structure to match the research question in a trial with a well-defined target of estimation, supporting specific clinical decisions. Adherence to this framework can help improve the quality of data collection, analysis and reporting of PROs in SATs and thereby increase end-users’ insight and confidence in their results, impacting decision making in clinical practice.

## Supplementary Information

Supplementary Material 1.

Supplementary Material 2.

## Data Availability

The data that support the findings of this study are available from Pfizer, Inc., but restrictions apply to the availability of these data, which were used under license for the current study, and so are not publicly available. Data are however available from the authors upon reasonable request and with permission of Pfizer, Inc.

## Abbreviations

ALK: Anaplastic lymphoma kinase
EMA: European Medicines Agency
FDA: U.S. Food and Drug Administration
GEE: Generalized Estimating Equations
LMM: Linear mixed model
MAR: Missing at random
PRO: Patient-reported outcome
QoL: Quality of life
SAT: Single-arm trial
TD: Treatment discontinuation
PD: Progression of the disease
