# Supplementary material for: The role of the estimand framework in the analysis of patient-reported outcomes in single-arm trials: a case study in oncology
Source: BMC Med Res Methodol. 2024 Nov 23;24:290. doi: 10.1186/s12874-024-02408-x (PMC11585159; doi:10.1186/s12874-024-02408-x)
Supplement: Supplementary file 1 — Supplementary Material 1. [file 12874_2024_2408_MOESM1_ESM.docx]

APPENDIX: The role of the estimand framework in the analysis of patient-reported outcomes in single-arm trials: a case study in oncology

# Supplement on missing data imputation

## Motivation for our imputation model

In this case study, we intended to deal with the issue of missing PRO data separately from the illustration of the estimand framework. We plan to elaborate on missing longitudinal PRO data in the presence of intercurrent events further in future work. The objective of our imputation for this case study was to keep it simple and create a single complete dataset that could serve as an example. We therefore used a single imputation method here, acknowledging that a multiple imputation method would be preferred in a real trial analysis because it accounts better for imputation uncertainty. Two focus points for the imputation were: (1) no imputation of QoL values after death; and (2) take the occurrence and timing of intercurrent events (death, progression of disease, treatment discontinuation) into account in the imputation of QoL.

Since the treatment was only available within the trial, treatment discontinuation was observed at some time point for all participants. A time of disease progression was not recorded for everyone. In this case study, we assumed that all death was disease-related (since participants had an advanced stage of lung cancer and the mortality rate was high), and if no PD was recorded before death, the date of death was set as the PD date. Those for whom no PD was recorded and who were censored for overall survival, were censored for PD at the overall survival censoring date.

In the available data, we summarized the PRO measurements backwards from death and censoring, setting the clock to zero at death or censoring. We found that on average, PRO scores dropped in the last five cycles before death, whereas no such drop was observed before censoring. The progression of disease and the decision to discontinue treatment may be related to patients’ QoL trajectories as well. We therefore assumed that the time-distance to death and other intercurrent events was possibly relevant to the missing PROs at each cycle.

## Imputation model specification

We imputed missing PRO data using single imputation until cycle 40, under the assumption that the PROs at each cycle were missing at random conditional on the cycle number, available QoL measurements at other cycles, death, PD and TD, and the time until these events. We used a linear mixed model with a random patient-intercept. Variables included were the cycle number (forward time scale), the number of cycles till death/PD/TD (backward time scales), time-varying indicators of whether PD/TD had occurred, and constant indicators of whether PD/death was observed or censored. Splines were applied for all backward time variables. We also included an interaction between the cycles till death/PD and the corresponding censoring indicators. This means that we assumed the time-relation between QoL and death/PD to be different from the time-relation between QoL and censoring (for survival and PD, respectively).

Our method to address missing PROs is informed by the occurrence and timing of ICEs, which may be observed after the missing PRO was planned to be measured. For this reason, direct likelihood-based methods where a mixed model is used for the implicit imputation and analysis of PROs at the same time would not be appropriate. While the information on (future) ICEs may be informative for the missing values, conditioning on future values in an analysis model would not target the estimand of interest. Therefore, to take ICE information into account, we opted to handle missing PRO values using a missing data model that was separate from the substantive analysis model.

Specifically, our imputation model was coded in R as follows:

lmer(QL2 ~ (1|patid) + as.factor(cycleno) + rms::rcs(cycles_till_PD,nk=4, knots = c(1,4, 9,20))*PD_observed + rms::rcs(cycles_till_TD,nk=4, knots = c(1,4, 9,20)) + rms::rcs(cycles_till_end,nk=4, knots = c(1,4, 9,20))*death_observed + PD_yet + TD_yet, …)

Patient-specific predictions from this model were used to impute missing QoL values. In our subsequent analyses, we assumed non-informative censoring in our analyses, as the majority of censoring was administrative censoring. In cases where the assumption of uninformative censoring is unlikely to hold, reweighing techniques based on the inverse probability of censoring might be applied.

## Results

The PRO means are slightly lower in the imputed dataset than in the available data. This was to be expected, as a large proportion of PROs were missing shortly before death, and in those which were available, we noted a drop before death (Figure S1). This drop was modelled in the imputation, leading to a larger proportion of lower QoL values in later cycles in the imputed dataset. Patients in a worse disease stage may have more intermittent missing PROs as well.


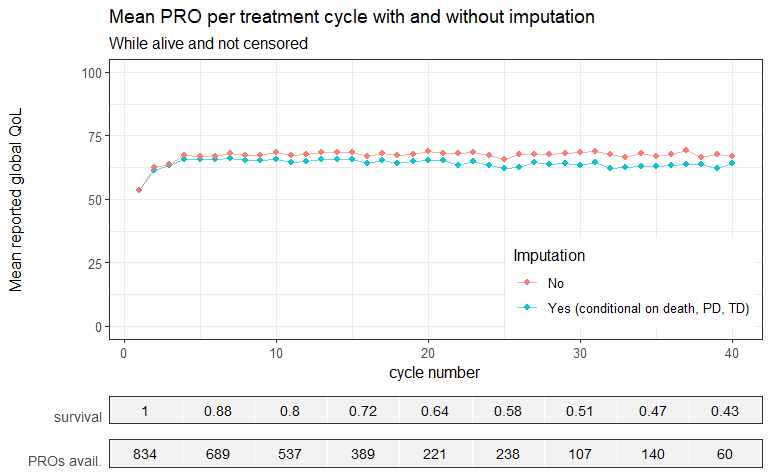


Figure S1. Mean reported global quality of life at each cycle, within the available data and within the dataset where missing values were imputed. All means are while alive and not censored.

# Applying the estimand framework to PROs in a single-arm trial: a review of possible choices

## Defining the variable of interest

### The absolute (numerical or ordinal) values of the PRO

Analyzing the absolute numerical PRO values at (a) prespecified time(s) is most straightforward and avoids information loss in outcome post-processing(1).

### Magnitude of change from baseline

The PRO change from baseline subtracts a person’s baseline value from their absolute PRO value at each cycle. With this choice of variable, we must consider ceiling and floor effects, since most PROs have a maximum and minimum possible value. Patients starting at the maximum (mininum) value can never have a positive (negative) change from baseline. Furthermore, if a clinically important change is defined on the individual patient level, patients whose baseline value is closer to the maximum/minimum than this change, can never experience such change (in the positive/negative direction, respectively), regardless of the effect of treatment.

When the baseline PRO is predictive for survival, trends in change from baseline scores in those alive may reflect the selection process due to death on the baseline values rather than an effect of the treatment on the PROs over time. Also, change scores have been shown not to generally estimate the intended causal effect in observational data(2), depending on the causal relation between the baseline score and later scores. In addition, regression to the mean and measurement error may affect observed change scores. Finally, the analysis of change scores is known to be statistically inefficient compared to an analysis of covariance with the baseline score as a covariate(3).

### Responder/non-responder classification

A third potential choice of variable is to classify patients as responders or non-responders—overall or at each cycle. For example, patients could be classified as ‘responder’ when their QoL values reach some threshold or increase by some amount.

A patient’s QoL reaching a threshold or having improved a certain amount from baseline are not necessarily definitive events; patients may deteriorate after having improved and vice versa. Classifying patients as overall responder or non-responder does not reflect this aspect of the individual patterns over time. When response is defined by a minimum improvement from baseline, ceiling and bottom effects may apply as well.

In addition, when defining overall responders, it is important to incorporate a relevant time-window in which the response must have occurred. Depending on the analysis, bias may arise when patients are more likely to become responders when they remain alive longer. Still, missing PRO values can lead to the timing of response being unknown. Another consideration is that any type of responder classification may create an artificial dichotomy between patients whose original QoL patterns are not very different qualitatively(4,5).

### Time to event: time to deterioration and time to response

When response or deterioration are definitive events, or when the time until the first such event is meaningful, a time-to-event variable may be of interest. Time till deterioration endpoints for PROs may be familiar to trialists in oncology, because of their analogy with progression free survival (PFS) as a clinical endpoint.

The considerations for responder classification outlined above apply here as well, in particular the possibly non-definitive nature of response and deterioration. An additional complication in time-to-event analysis of PROs is that a drop or rise in the outcome is recorded at the next time a questionnaire is filled in (if the PRO has not changed again). Therefore, the exact time of response or deterioration is only known to lie between two assessments (interval censoring). In a recent paper, Fiero et al.(6) suggest an analysis at pre-specified relevant timepoints rather than a time-to-event approach.

## Population level summary

When the aim of the study is descriptive, the mean variable in each cycle may be a suitable population level summary of the absolute values of the PRO and changes from baseline. For responder classification, the proportion of responders in relevant cycles may be of interest. A time-to-event variable may be summarized with an estimate of the probability of the event over time. Sometimes a single summary value is of interest, e.g., when external comparisons are made for benchmarking. The mean variable at one or several prespecified, relevant time points is then another option.

## Strategies for dealing with intercurrent events and death

The ICH E9 (R1)^4^ defines intercurrent events (IEs) as events that occur after the start of a trial and affect the presence and/or interpretability of observed outcome values. We focus on three such events: death, disease progression (PD), treatment discontinuation (TD).

The estimand framework suggests five strategies for handling intercurrent events in an analysis. The chosen strategy should reflect the study aim, as it will determine the interpretation of the results of the analysis.

### ‘While the intercurrent event has not yet occurred’–strategy

This strategy entails that we aim to obtain our population level summary at each relevant time point for those patients who are still without the IE at that time point. For death, for instance, this means we are interested in the PROs of those patients who are still alive (while alive strategy).

### Composite strategy

Sometimes the outcome of interest and the intercurrent event are combined into a different, composite outcome. An example is QoL measured with EQ-5D, where a value of 0 is defined for QoL after death. Another example is a time-till-deterioration endpoint where deterioration is defined as QoL below a certain value or death.

### Hypothetical strategy

A third strategy is to estimate the population level summary in a hypothetical world where the intercurrent event could be postponed until after the end of the trial for all patients. A corresponding example estimand would be the mean QoL at cycle 10, if no one would die or discontinue treatment before cycle 10.

### Treatment policy strategy

A treatment policy strategy ignores the intercurrent event in the analysis. This reflects the intention-to-treat principle: the treatment effect is estimated based on assigned treatment at study onset, regardless of what happens afterwards. The estimated effect aims to represent the effect of a treatment policy. After death, however, PRO values do not exist, hence death cannot be ignored in the analysis^4^.

### Principal stratum strategy

A principal stratum is a subgroup of patients who would or who would not have experienced the event under either of the treatments in a study. For death, a principal stratum might be those patients who would have survived during the study, irrespective of treatment received. A corresponding estimand would be the difference in mean outcome for treatment A vs B, in those surviving the study, regardless of their treatment assignment. A patient’s intercurrent events can only be observed for the treatment arm they were actually in, so the principal stratum strategy requires counterfactual information about whether they would have had the event, had they been assigned to the other treatment arm. Since the focus of this report is on single-arm trials, where there is only one treatment in the study, we did not apply a principal stratum strategy.

# Supplementary figures and tables


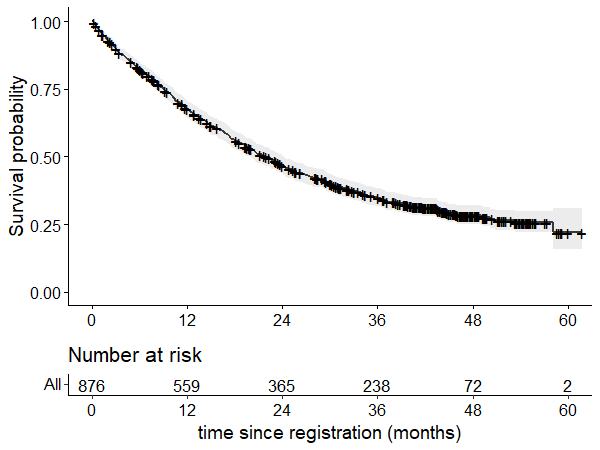


Figure S2. Kaplan-Meier estimate of the probability of overall survival in our case study cohort.

|  | Overall (N=876) |
| --- | --- |
| Sex |  |
| Female | 501 (57.2%) |
| Male | 375 (42.8%) |
| Age (years) |  |
| Mean (SD) | 53.0 (12.4) |
| No. of previous drug treatments |  |
| 1 | 181 (20.7%) |
| 2 | 323 (36.9%) |
| 3 | 189 (21.6%) |
| 4 | 91 (10.4%) |
| 5 | 49 (5.6%) |
| 6 | 22 (2.5%) |
| > 6 | 21 (2.4%) |
| ECOG performance status at baseline |  |
| 0 | 249 (28.4%) |
| 1 | 480 (54.8%) |
| 2 | 119 (13.6%) |
| 3 | 28 (3.2%) |
| Days to treatment discontinuation |  |
| Median [Min, Max] | 327 [1.00, 1690] |
| Days to first disease progression |  |
| Median [Min, Max] | 249 [6.00, 1550] |
| Missing | 228 (26.0%) |
| Death observed |  |
| No | 300 (34.2%) |
| Yes | 576 (65.8%) |
| Objective tumor response observed |  |
| No | 411 (46.9%) |
| Yes | 465 (53.1%) |

Table S1. Demographical and clinical characteristics of the patients included in our case study.


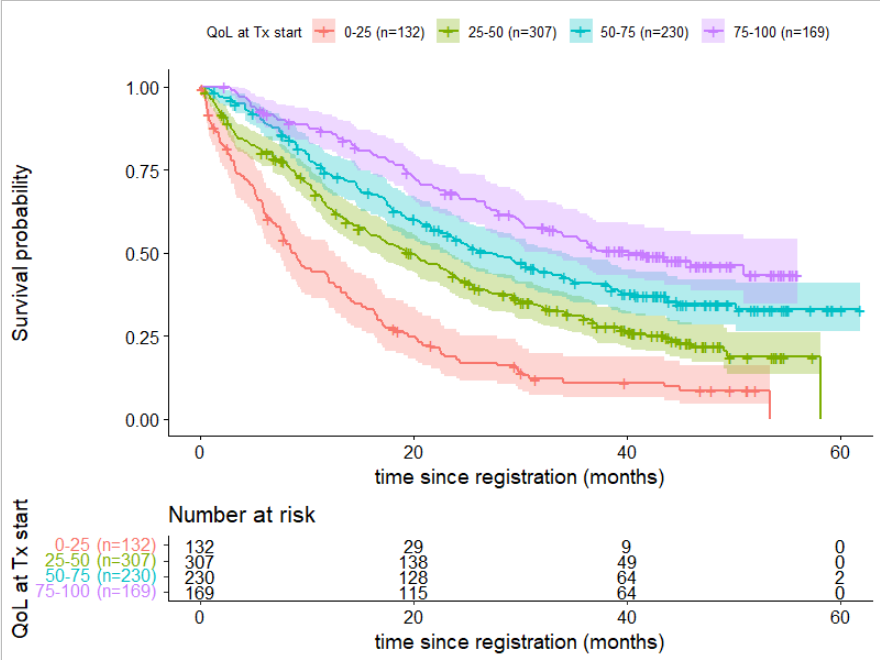


Figure S3. Kaplan-Meier estimates of overall survival in our case study cohort, stratified by QoL at the start of protocol treatment (in participants who reported QoL in their first treatment cycle). Participants were stratified based on four intervals of baseline QoL values: [0, 25], (25, 50], (50, 75] and (75, 100]. The shaded regions represent 95% confidence intervals.

Figure S4. Distributions of the time between the last available PRO measurement and death (A), censoring (B), treatment discontinuation (C) and progression of disease (D), respectively


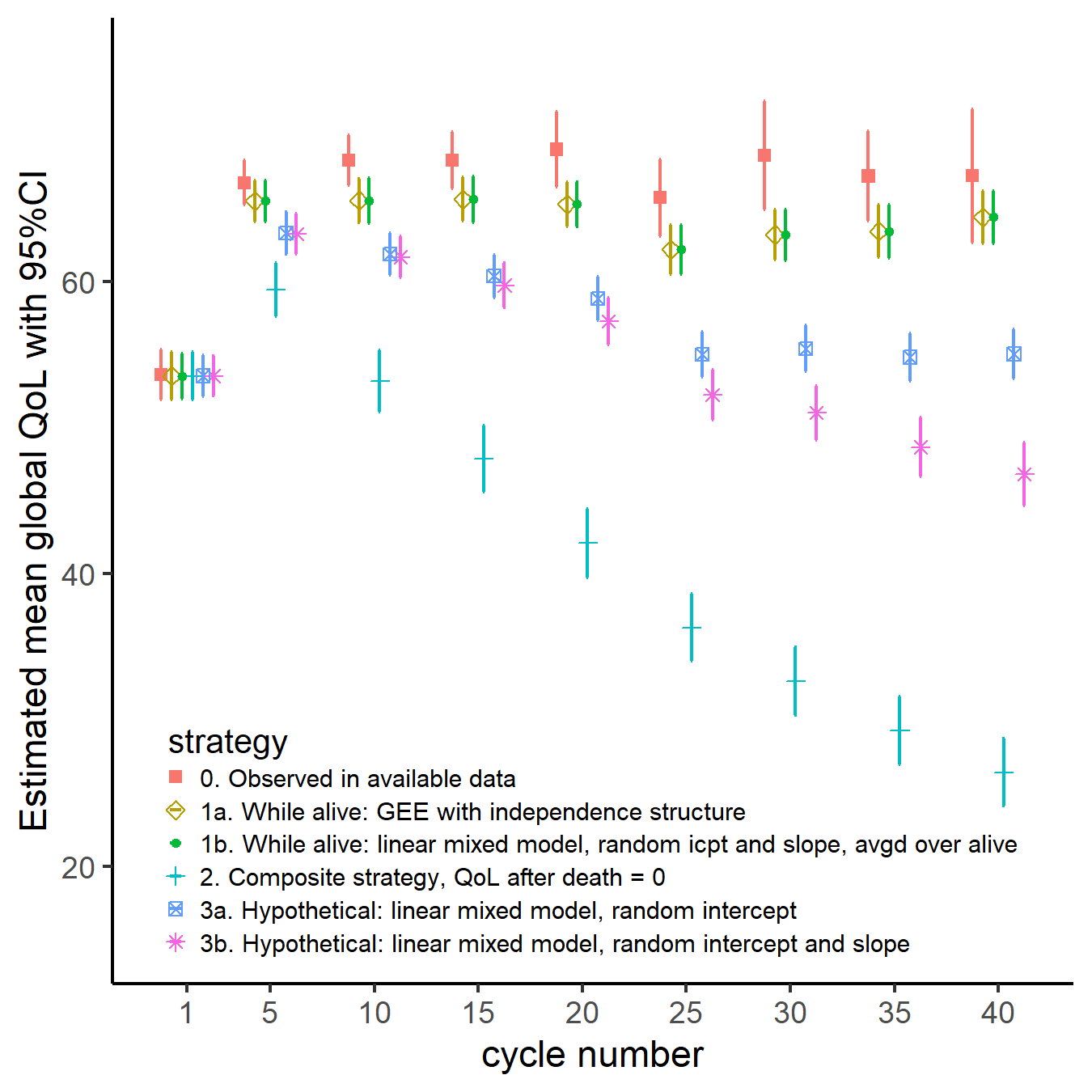


Figure S5. Estimated mean global QoL and corresponding 95% CIs at the first and every fifth cycle, for a while alive, composite and hypothetical strategy to deal with death. No major differences in CI width occurred between the various analysis methods used.


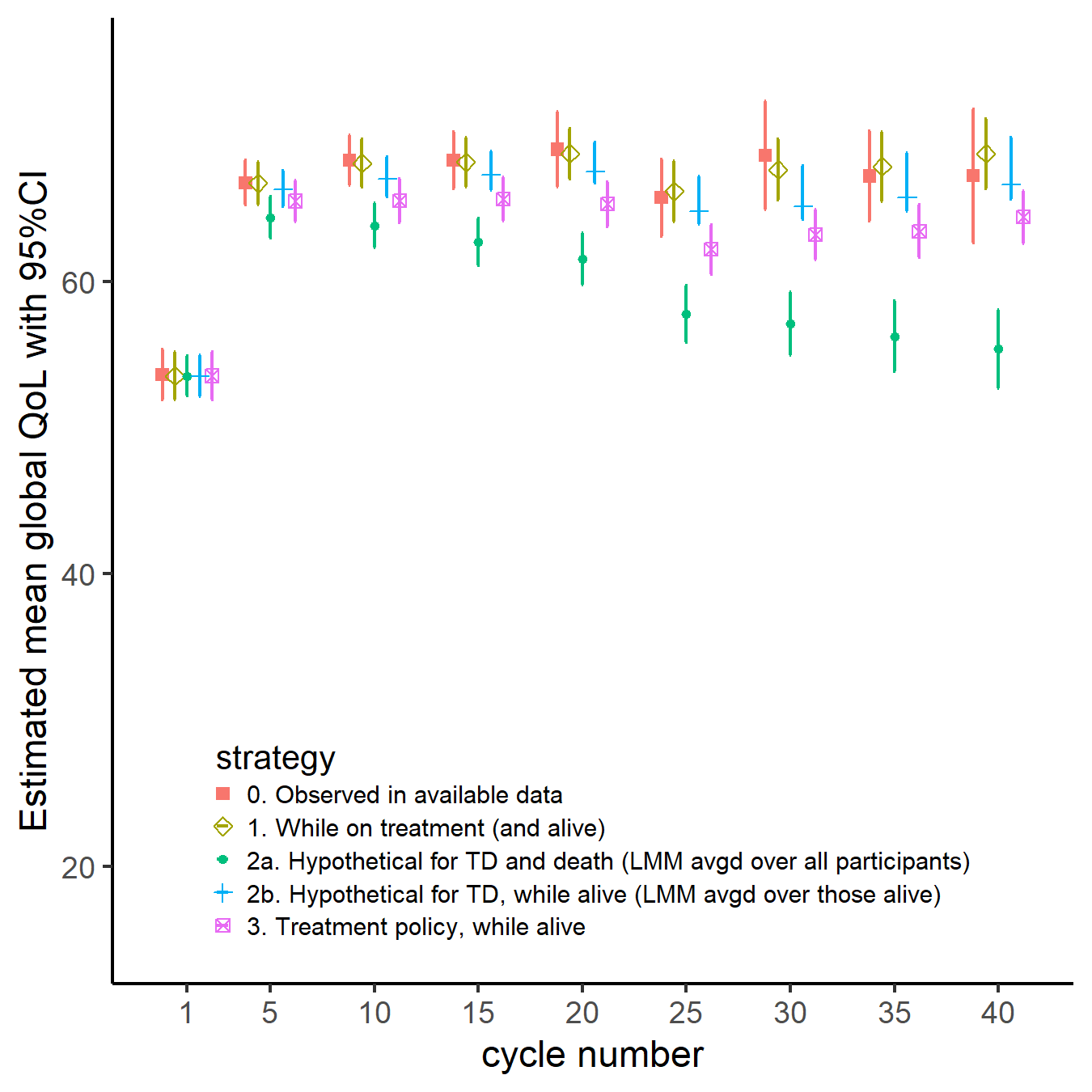


Figure S6. Estimated mean global QoL and corresponding 95% CIs at the first and every fifth cycle, for various strategies to deal with treatment discontinuation.


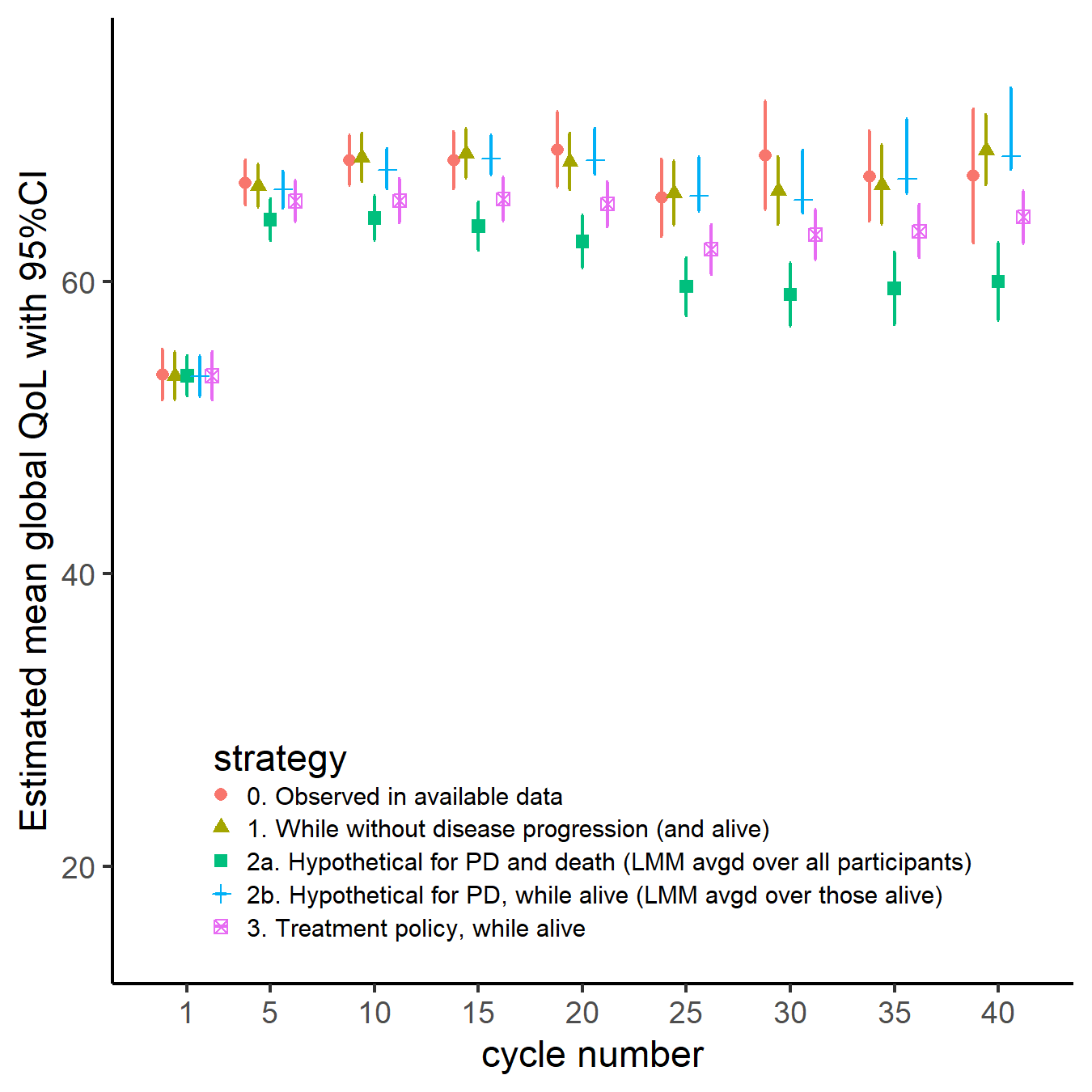


Figure S7. Estimated mean global QoL and corresponding 95% CIs at the first and every fifth cycle, for various strategies to deal with disease progression.

# References

1. Collister D, Bangdiwala S, Walsh M, Mian R, Lee SF, Furukawa TA, et al. Patient reported outcome measures in clinical trials should be initially analyzed as continuous outcomes for statistical significance and responder analyses should be reserved as secondary analyses. J Clin Epidemiol. 2021 Jun;134:95–102.

2. Tennant PWG, Arnold KF, Ellison GTH, Gilthorpe MS. Analyses of ‘change scores’ do not estimate causal effects in observational data. Int J Epidemiol. 2022 Oct 13;51(5):1604–15.

3. Vickers AJ. The use of percentage change from baseline as an outcome in a controlled trial is statistically inefficient: a simulation study. BMC Med Res Methodol. 2001 Dec;1(1):6.

4. Cappelleri JC, Chambers R. Addressing Bias in Responder Analysis of Patient-Reported Outcomes. Ther Innov Regul Sci. 2021 Sep;55(5):989–1000.

5. Cappelleri JC. Further reduction in statistical power for responder analysis of patient-reported outcomes with measurement error. J Clin Epidemiol. 2021 Dec;140:200–1.

6. Fiero MH, Roydhouse JK, Bhatnagar V, Chen TY, King-Kallimanis BL, Tang S, et al. Time to deterioration of symptoms or function using patient-reported outcomes in cancer trials. Lancet Oncol. 2022 May;23(5):e229–34.
