# Supplementary material for: The role of the estimand framework in the analysis of patient-reported outcomes in single-arm trials: a case study in oncology
Source: BMC Med Res Methodol. 2024 Nov 23;24:290. doi: 10.1186/s12874-024-02408-x (PMC11585159; doi:10.1186/s12874-024-02408-x)
Supplement: Supplementary file 2 — Supplementary Material 2. [file 12874_2024_2408_MOESM2_ESM.html]

The role of the estimand framework in the analysis of patient-reported outcomes in single-arm trials: a case study in oncology – ANALYSIS CODE


# The role of the estimand framework in the analysis of patient-reported outcomes in single-arm trials: a case study in oncology – ANALYSIS CODE

#### Doranne Thomassen

#### 2024-03-27

- Packages
  used
- Descriptive analyses
  - Plot
    cumulative availability of PRO data per cycle (Figure 1)
  - Make
    a table of QoL availability to provide with estimated mean QoL
  - Make
    a table of overall survival to provide with estimated mean QoL
- Applying the
  estimand framework to our case study
  - Defining the
    variable of interest (Figure 2)
    - Mean of the numerical PRO
    - Mean change from baseline
    - Responder/non-responder
      classification
    - Make one big
      plot
  - Strategies
    for dealing with intercurrent events and death
    - Single
      imputation of QoL while alive, taking into account the timing of
      intercurrent events
    - create
      ‘survival tables’ for PD and TD to provide with estimated mean
      QoL
    - Estimating
      mean QoL over time under different strategies to deal with death (Figure
      3)
    - Estimating
      mean QoL over time under different strategies to deal with treatment
      discontinuation (Figure 4)
    - Estimating
      mean QoL over time under different strategies to deal with disease
      progression (Figure 5)

# Packages used

```
require(grid)
require(gridExtra)
require(patchwork)
require(survival)
require(survminer)
require(ggplot2)
require(reshape2)
require(Hmisc)
require(patchwork)
require(glmtoolbox)
require(geepack)
require(emmeans)
require(lmeresampler)
```

# Descriptive analyses

We assume here that the data are already loaded. ‘QoL\_dataset’ is a
longitudinal dataset with QoL measurements in long format, i.e., each
row contains measured QoL at one cycle (and possibly other
measurements/data at that cycle) and a patient identifier ‘pat.id’
indicating to which patients the measurement belongs. Each patient will
have several corresponding rows, one for each cycle number where QoL was
planned to be measured.

‘patinfo’ is a dataset with one row for each patient, which contains
baseline characteristics as well as important event dates and event
indicators (randomization, start treatment, stop treatment, intercurrent
events, death/censoring, …).

The first day of the first treatment cycle is taken as baseline in
this analysis.

TD = treatment discontinuation; PD = disease progression

## Plot cumulative availability of PRO data per cycle (Figure 1)

```
n <- nrow(patinfo)
QoL_dataset$QoLmissing <- as.numeric(is.na(QoL_dataset$QoL))
QoL_dataset$QoLnonmissing <- as.numeric(!is.na(QoL_dataset$QoL))
patinfo$cycle_EOT <- floor(as.numeric(patinfo$date_stopTx - patinfo$date_startTx)/21)+1
patinfo$cycle_death <- floor(as.numeric(patinfo$death)/21)+1

# Count patients on/off treatment and with/without QoL measurements available for each cycle
max.cycleno = max(as.numeric(QoL_dataset$cycleno), na.rm = TRUE)
state.dat <- data.frame(pat.id=rep(patinfo$pat.id, each=max.cycleno),
                        cycleno = rep((1:max.cycleno),n))
state.dat$state <- character(nrow(state.dat))

for(p in (1:n)){
  for(c in (1:max.cycleno)){
    # Check if censored for overall survival
    if((patinfo$days_till_death_or_cens[p] <= 21*(c-1)+1) & patinfo$death[p]==0){
      state.dat$state[((p-1)*max.cycleno)+c] = "5. survival status unknown"
    }
    # Check if deceased
    else if ((patinfo$days_till_death_or_cens[p] <= 21*(c-1)+1) & patinfo$death[p]==1){
      state.dat$state[((p-1)*max.cycleno)+c] = "6. deceased"}
    # Check if QoL available in QoL dataset for this patient
    else if(c %in% QoL_dataset$cycleno[which(QoL_dataset$pat.id==patinfo$pat.id[p] & (!is.na(QoL_dataset$QoL)))]){
      # Check if on treatment
      if(c <= patinfo$cycle_EOT[p]){  
        state.dat$state[((p-1)*max.cycleno)+c] = "1. QoL available and on treatment"
      }
      else{
        state.dat$state[((p-1)*max.cycleno)+c] = "2. QoL available and off treatment"}
    }
    # If QoL not available...
    else{
      # Check if on treatment
      if(c <= patinfo$cycle_EOT[p]){
        state.dat$state[((p-1)*max.cycleno)+c] = "3. QoL not available and on treatment"
      }
      else{
        state.dat$state[((p-1)*max.cycleno)+c] = "4. QoL not available and off treatment"
      }
    }
  }
}

state.dat$count <- rep(1,nrow(state.dat))
#aggregate(count~cycleno+state, data=state.dat, FUN = "sum")

state.dat$state <- factor(state.dat$state,
                                levels = c("6. deceased",
                                           "5. survival status unknown",
                                           "4. QoL not available and off treatment",
                                           "3. QoL not available and on treatment",
                                           "2. QoL available and off treatment",
                                           "1. QoL available and on treatment"
                                    ))

ggplot(data=state.dat, aes(fill=state, y=count, x=as.numeric(cycleno))) +
    geom_bar(position="stack", stat="identity", alpha=1)+
  theme_bw()+
  ggtitle("Number of patients with PROs available per treatment cycle")+
  xlab("cycle number")+
  geom_vline(xintercept = 40, linetype="dashed", color="grey40")

ggsave(filename="number_stages.pdf", width = 8, height = 4)
```

## Make a table of QoL availability to provide with estimated mean QoL

```
 prop.avail <- function(X){
  prop <- sum(as.numeric(X=="3. QoL available and off treatment" | X=="1. QoL available and on treatment"))
  return(prop)
}

QoL.avail <- aggregate(state~cycleno, data = state.dat[,c("cycleno", "state")], FUN = prop.avail)
#summary(as.factor(summary(as.factor(state.dat$pat.id[which(state.dat$state=="QoL available and off treatment")]))))


QoL.avail <- QoL.avail[QoL.avail$cycleno %in% c(1,5,10,15,20,25,30,35,40),] 
QoL.avail[1,"cycleno"]=0
#QoL.avail[1, "state"]=876
###
     


rownames(QoL.avail) <- seq(from=0, to=40, by=5)
tab <- gridExtra::tableGrob(t(QoL.avail$state))
tab$widths <- unit(rep(1, ncol(tab)), "null")
tab$heights <- unit(rep(1, nrow(tab)), "null")

is_text <- vapply(tab$grobs, inherits, logical(1), "text")
tab$grobs[is_text] <- lapply(tab$grobs[is_text], function(text) {
  text$gp$fontsize <- 8
  text
})


p4 <- ggplot() +
  annotation_custom(tab) +
  scale_y_discrete(breaks = (c("PROs avail.")), 
                   limits = c(("PROs avail."), ""))+
  theme(axis.ticks.y = element_blank(),
        text = element_text(size=9.5))
```

## Make a table of overall survival to provide with estimated mean QoL

```
KM <- round(survfit(Surv(time=cycle_death,event=death)~1, data=patinfo)$surv[1:40], digits=2)

surviving <- data.frame(KM)
surviving$cycleno <- 1:40
surviving <- surviving[surviving$cycleno %in% c(1,5,10,15,20,25,30,35,40),] 
surviving[1,"cycleno"]=0
surviving[1, "KM"]=1


rownames(surviving) <- seq(from=0, to=40, by=5)
tab <- gridExtra::tableGrob(t(surviving$KM))
tab$widths <- unit(rep(1, ncol(tab)), "null")
tab$heights <- unit(rep(1, nrow(tab)), "null")

is_text <- vapply(tab$grobs, inherits, logical(1), "text")
tab$grobs[is_text] <- lapply(tab$grobs[is_text], function(text) {
  text$gp$fontsize <- 8
  text
})


p3 <- ggplot() +
  annotation_custom(tab) +
  scale_y_discrete(breaks = (c("survival")), 
                   limits = c(("survival"), ""))+
  theme(axis.ticks.y = element_blank(),
        text = element_text(size=9.5))
```

# Applying the estimand framework to our case study

## Defining the variable of interest (Figure 2)

### Mean of the numerical PRO

```
QoL_means.dat <- aggregate(QoL~cycleno, data=QoL_dataset[,c("QoL", "cycleno")], FUN = function(x) c(mean = mean(x), se.mean=sqrt(var(x)/length(x))))
QoL_means.dat$QoL_mean = QoL_means.dat$QoL[,"mean"]
QoL_means.dat$QoL_se.mean = QoL_means.dat$QoL[,"se.mean"]
QoL_means.dat$CIupp = QoL_means.dat$QoL_mean+1.96*QoL_means.dat$QoL_se.mean
QoL_means.dat$CIlow = QoL_means.dat$QoL_mean-1.96*QoL_means.dat$QoL_se.mean


QoLmeans <- ggplot(data = QoL_means.dat, aes(x = as.numeric(cycleno), y = QoL_mean))+
  geom_point(color="black", fill="black")+
  geom_errorbar(aes(ymin=CIlow, ymax=CIupp), width=.2,
                position=position_dodge(0.05))+
  scale_x_continuous(breaks = c(0,5,10,15,20,25,30,35,40),
                     expand = c(0,0.5)) +
  theme_bw()+
  xlim(c(0,40))+
  ylim(c(0,100))+
  ylab("Mean global QoL")+
  xlab("cycle number")#+
  #ggtitle("Mean PRO values per treatment cycle", subtitle = "Within available measurements only")

#ggsave(filename="QoLmeans.pdf", width = 8, height = 4)
```

### Mean change from baseline

```
QoL_dataset$QoLdiff <- QoL_dataset$QoL - QoL_dataset$baselineQoL


QoL_diffmeans.dat <- aggregate(QoLdiff~cycleno, data=QoL_dataset[,c("QoLdiff", "cycleno")], FUN = function(x) c(mean = mean(x), se.mean=sqrt(var(x)/length(x))))
QoL_diffmeans.dat$QoLdiff_mean = QoL_diffmeans.dat$QoLdiff[,"mean"]
QoL_diffmeans.dat$QoLdiff_se.mean = QoL_diffmeans.dat$QoLdiff[,"se.mean"]
CIupp <- QoL_diffmeans.dat$QoLdiff_mean +1.96*QoL_diffmeans.dat$QoLdiff_se.mean
CIlow <- QoL_diffmeans.dat$QoLdiff_mean -1.96*QoL_diffmeans.dat$QoLdiff_se.mean
QoL_diffmeans.dat <- cbind(QoL_diffmeans.dat, CIupp, CIlow)

QoLdiffmeans <- ggplot(data = QoL_diffmeans.dat[], aes(x = as.numeric(cycleno), y = QoLdiff_mean))+
  geom_point(color="black", fill="black")+
  geom_errorbar(aes(ymin=CIlow, ymax=CIupp), width=.2,
                position=position_dodge(0.01))+
  theme_bw()+
  xlim(c(0,40))+
  ylim(c(-50,50))+
  #geom_hline(yintercept=c(-10,10), linetype="dashed")+
  #ggtitle("Mean difference from baseline in PROs per treatment cycle", subtitle = "Whithin available measurements only")+
  ylab("Mean change from baseline")+
  xlab("cycle number")
#ggsave(filename="QoLdiffmeans.pdf", width = 8, height = 4)
```

### Responder/non-responder classification

```
QoL_diffprops.dat <- aggregate(QoLdiff~cycleno, data=QoL_dataset[,c("QoLdiff", "cycleno")], 
                               FUN = function(x) {prop = sum(as.numeric(x>10))/length(x)
                                                  se.prop=sqrt((prop*(1-prop))/length(x))
                                                  return(c(prop=prop, se.prop=se.prop))}
                               )
QoL_diffprops.dat$QoLdiff_prop = QoL_diffprops.dat$QoLdiff[,"prop"]
QoL_diffprops.dat$QoLdiff_se.prop = QoL_diffprops.dat$QoLdiff[,"se.prop"]
CIupp <- QoL_diffprops.dat$QoLdiff_prop +1.96*QoL_diffprops.dat$QoLdiff_se.prop
CIlow <- QoL_diffprops.dat$QoLdiff_prop -1.96*QoL_diffprops.dat$QoLdiff_se.prop
QoL_diffprops.dat <- cbind(QoL_diffprops.dat, CIupp, CIlow)

QoL_prop_responder <- ggplot(data = QoL_diffprops.dat, aes(x = as.numeric(cycleno), y = 100*QoLdiff_prop))+
  geom_point(color="black", fill="black")+
  geom_errorbar(aes(ymin=100*CIlow, ymax=100*CIupp), width=.2,
                position=position_dodge(0.05))+
  theme_bw()+
  #ggtitle("Percentage of patients with at least 10 points increase from baseline in QoL", subtitle = "In alive patients with available QoL values")+
  xlim(c(0,40))+
  ylim(c(0,100))+
  ylab("% of responders")+
  xlab("cycle number")

#ggsave(filename="QoLdiffprops.pdf", width = 8, height = 4)
```

### Make one big plot

```
#Make one big plot
QoLmeans + QoLdiffmeans + QoL_prop_responder + p3 + p4 + plot_layout(ncol = 1, heights=c(9,9,9,1, 1))
```

## Strategies for dealing with intercurrent events and death

### Single imputation of QoL while alive, taking into account the timing of intercurrent events

See appendix for details, we plan to write a separate, more technical
paper on the imputation of longitudinal PROs but for the purpose of
illustration in this case study we kept it relatively simple.

```
patinfo$cycle_eoFU <- as.numeric(floor(patinfo$days_till_death_or_cens/21)+1)
patinfo$cycle_eoTx <- as.numeric(floor((patinfo$date_stopTx-patinfo$date_startTx)/21)+1)
patinfo$cycle_PD <- as.numeric(floor((patinfo$date_PD-patinfo$date_startTx)/21)+1)

#create vector of unique patient IDs in the QoL_dataset data
pat_numbers <- sort(unique(QoL_dataset$pat.id), decreasing=FALSE)

maxcycles <- max(patinfo$cycle_eoFU)

#create empty grid
QoL_imp1 <- data.frame(pat.id=as.numeric(rep(pat_numbers, each=maxcycles)),
                       cycleno=rep(1:maxcycles, times=length(pat_numbers)))

#fill in relevant patient info
QoL_imp1 <- merge(QoL_imp1, patinfo[,c("pat.id", "death", "cycle_eoFU", "cycle_eoTx", "cycle_PD")], by=c("pat.id"), all = TRUE, sort=TRUE)
QoL_imp1$died <- QoL_imp1$death
QoL_imp1$lastcycle_no <- QoL_imp1$cycle_eoFU
QoL_imp1$lastcycle <- QoL_imp1$cycleno==QoL_imp1$lastcycle_no
QoL_imp1$cycles_till_end <- QoL_imp1$lastcycle_no- QoL_imp1$cycleno

#prep QoL variable: remove NAs when there is another available measurement within the same cycle and take averages if there are >1 available measurements per cycle
QoL_dataset$cycleno <- as.numeric(QoL_dataset$cycleno)
QoL_avgd <- aggregate(QoL ~ pat.id + cycleno, data=QoL_dataset, FUN=function(x){mean(x, na.rm=TRUE)}, drop=FALSE, na.action = na.omit)

#Fill in QoL values into the new grid
QoL_imp1 <- merge(QoL_imp1, QoL_avgd, by=c("pat.id", "cycleno"), all = TRUE, sort=TRUE)

#create last measurement indicator
QoL_imp1$lastmeas <- numeric(nrow(QoL_imp1))
for(i in 1:pat_numbers){
  QoL_imp1$lastmeas[which(QoL_imp1$pat.id==i)] = max(QoL_imp1$cycleno[which(QoL_imp1$pat.id==i & !is.na(QoL_imp1$QoL))])
}

QoL_grid <- QoL_imp1

##Create a dataframe in long format for imputation
#remove everything after cycle 40
maxcycles <- 40
QoL_grid <- QoL_grid[-which(QoL_grid$cycleno>maxcycles),]

QoL_grid <- subset(QoL_grid, select = -c(`lastcycle`))

#Remove all rows after death/censoring
QoL_grid <- QoL_grid[-which(QoL_grid$cycleno>QoL_grid$lastcycle_no),]

#------------- coding the intercurrent events -------------------------------
#Add indicator of disease progession ---> ASSUMPTION: All death is disease-related in this study. So those who died get PD=1 even if no date of PD recorded
QoL_grid$PD <- as.numeric(!is.na(QoL_grid$cycle_PD) | QoL_grid$died==1)

#Add time distance to PD with a mininum of 0 and which equals 0 for those in whom we do not observe PD
QoL_grid$cycles_till_PD <- as.numeric(QoL_grid$cycle_PD-QoL_grid$cycleno)
QoL_grid$cycles_till_PD[which(is.na(QoL_grid$cycles_till_PD) & QoL_grid$died==1)] <- QoL_grid$cycles_till_end[which(is.na(QoL_grid$cycles_till_PD) & QoL_grid$died==1)]
QoL_grid$cycles_till_PD[which(is.na(QoL_grid$cycles_till_PD) & QoL_grid$died==0)] <- 0
QoL_grid$cycles_till_PD[which(QoL_grid$cycles_till_PD < 0)] <- 0
QoL_grid$cycle_PD[which(is.na(QoL_grid$cycle_PD))] <- QoL_grid$lastcycle_no[which(is.na(QoL_grid$cycle_PD))]

#Time-varying indicator of whether PD has occurred yet in those who will get it
QoL_grid$PD_yet <- as.numeric(QoL_grid$cycles_till_PD <=0 & QoL_grid$PD==1)

#Add time distance to Treatment Discontinuation with a minimum of 0
#TD is observed in everyone, since one can only be on the treatment while in the study
QoL_grid$cycles_till_TD <- as.numeric(QoL_grid$cycle_eoTx-QoL_grid$cycleno)
QoL_grid$cycles_till_TD[which(QoL_grid$cycles_till_TD < 0)] <- 0
QoL_grid$TD_yet <- as.numeric(QoL_grid$cycles_till_TD <=0)

# Add interactions
QoL_grid$cycleno_factor <- as.factor(QoL_grid$cycleno)
QoL_grid$diedxtime <- QoL_grid$died * QoL_grid$cycles_till_end
QoL_grid$PDxtime <- QoL_grid$PD * QoL_grid$cycles_till_PD


#-----Use flexible mixed model with splines AND interactions to impute missing QoL measurements
require(lme4)
imp_LMM5 <- lmer(QoL ~ (1|pat.id) + as.factor(cycleno) + rms::rcs(cycles_till_PD,4)*PD + rms::rcs(cycles_till_TD,4) + rms::rcs(cycles_till_end,4)*died + PD_yet + TD_yet,
                  data=QoL_grid)

QoL_grid$pred_LMM5 <- predict(imp_LMM5, newdata=QoL_grid, re.form=NULL, allow.new.levels=TRUE)
QoL_grid$QoL[which(is.na(QoL_grid$QoL))] <- QoL_grid$pred_LMM5[which(is.na(QoL_grid$QoL))]

# Calculate means in those alive in the imputed vs unimputed data  
means_QoL_LMM5 <- aggregate(QoL~cycleno, data=QoL_grid, FUN = function(x){mean(x, na.rm=TRUE)})
impstrategies.dat <- data.frame(cycleno = 1:40,
                                  QoL_mean = means_QoL_LMM5$QoL[1:40],
                                  imp_method=rep("Yes (conditional on death, PD, TD)", 40))

impstrategies.dat <- rbind(impstrategies.dat,
                           data.frame(cycleno = 1:40,
                                  QoL_mean = QoL_means.dat$QoL_mean[1:40],
                                  imp_method=rep("No", 40))
)

#------PLOT-------------

imp_plot <- ggplot(data = impstrategies.dat, aes(x=cycleno, y=QoL_mean, color=imp_method, fill=imp_method))+
  geom_point(alpha=0.9)+
  geom_line(alpha=0.6)+
  #ylim(c(0,100))+
  ggtitle("Mean PRO per treatment cycle with and without imputation", subtitle = "While alive and not censored")+
  theme_bw()+
  ylim(c(0,100))+
  xlab("cycle number")+
  ylab("Mean reported global QoL")+
  labs(color="Imputation", fill="Imputation")+
  theme(legend.position = c(0.8, 0.2))

ggarrange(imp_plot, p3, p4, ncol = 1, heights = c(9,1,1), widths = c(10,1,1), align="v")

#------Merge imputed values into the full QoL dataset (with QoL=NA after death/censoring) and remove rows after cycle 40
QoL_imp2 <- merge(subset(QoL_imp1, select = -c(`QoL`)), QoL_grid[,c("QoL", "pat.id", "cycleno")], by=c("pat.id", "cycleno"), all = TRUE, sort=TRUE)
QoL_imp2 <- QoL_imp2[-which(QoL_imp2$cycleno > 40),]
```

### create ‘survival tables’ for PD and TD to provide with estimated mean QoL

```
# first for TD
patinfo$event_TD <- 1

#using kaplan meier for coding convenience here, but there is no censoring so estimates will just be the observed numbers
KM_TD <- round(survfit(Surv(cycle_eoTx, event_TD)~1, data=patinfo)$surv[1:40], digits=2)

ontrt <- data.frame(KM_TD)
ontrt$cycleno <- 1:40
ontrt <- ontrt[ontrt$cycleno %in% c(1,5,10,15,20,25,30,35,40),] 
ontrt[1,"cycleno"]=0
ontrt[1, "KM_TD"]=1


rownames(ontrt) <- seq(from=0, to=40, by=5)
tab <- gridExtra::tableGrob(t(ontrt$KM_TD))
tab$widths <- unit(rep(1, ncol(tab)), "null")
tab$heights <- unit(rep(1, nrow(tab)), "null")

is_text <- vapply(tab$grobs, inherits, logical(1), "text")
tab$grobs[is_text] <- lapply(tab$grobs[is_text], function(text) {
  text$gp$fontsize <- 8
  text
})


p_td <- ggplot() +
  annotation_custom(tab) +
  scale_y_discrete(breaks = (c("on treatment")), 
                   limits = c(("on treatment"), ""))+
  theme(axis.ticks.y = element_blank(),
        text = element_text(size=9.5))

#for PD
KM_PD <- round(survfit(Surv(cycle_PD_tab, event_PD)~1, data=patinfo)$surv[1:40], digits=2)

without_PD <- data.frame(KM_PD)
without_PD$cycleno <- 1:40
without_PD <- without_PD[without_PD$cycleno %in% c(1,5,10,15,20,25,30,35,40),] 
without_PD[1,"cycleno"]=0
without_PD[1, "KM_PD"]=1

rownames(without_PD) <- seq(from=0, to=40, by=5)
tab <- gridExtra::tableGrob(t(without_PD$KM_PD))
tab$widths <- unit(rep(1, ncol(tab)), "null")
tab$heights <- unit(rep(1, nrow(tab)), "null")

is_text <- vapply(tab$grobs, inherits, logical(1), "text")
tab$grobs[is_text] <- lapply(tab$grobs[is_text], function(text) {
  text$gp$fontsize <- 8
  text
})


p_pd <- ggplot() +
  annotation_custom(tab) +
  scale_y_discrete(breaks = (c("progression-free")), 
                   limits = c(("progression-free"), ""))+
  theme(axis.ticks.y = element_blank(),
        text = element_text(size=9.5))
```

### Estimating mean QoL over time under different strategies to deal with death (Figure 3)

Note that QoL\_imp2 is a QoL dataset in long format with rows for each
cycle from 1 through 40 for each patient. All missing QoL before
death/censoring has been imputed, so there is no missing QoL before
death/censoring in this data. For each patient, in the cycles after
death/censoring, QoL is set to NA in the data.

```
# OBSERVED MEANS ---------------------------------------
deathstrategies.dat <- data.frame(cycleno = 1:40, 
                                  QoL_mean = QoL_means.dat$QoL_mean[1:40],
                                  QoL_se.mean = QoL_means.dat$QoL_se.mean[1:40],
                                  strategy=rep("0. Observed in available data", 40))

# WHILE ALIVE -------------------------------------------
gee1 <- glmgee(formula = QoL ~ as.factor(cycleno), data=QoL_imp2, id=pat.id ,corstr = "independence")

gee_pred.means <- data.frame(cycleno = 1:40)
gee_pred.means$mean_QoL <- predict(gee1, newdata = gee_pred.means, se.fit = TRUE)[,"fit"]
gee_pred.means$se.mean_QoL <- predict(gee1, newdata = gee_pred.means, se.fit = TRUE)[,"se.fit"]
deathstrategies.dat <- rbind(deathstrategies.dat, 
                             data.frame(cycleno = 1:40,
                                        QoL_mean = gee_pred.means$mean_QoL,
                                        QoL_se.mean = gee_pred.means$se.mean_QoL,
                                        strategy=rep("1a. While alive: GEE with independence structure", 40)))

#COMPOSITE ------------------------------------------------
#impute a value of 0 for QoL after death
QoL_imp2_comp0 <- QoL_imp2
QoL_imp2_comp0$QoL[which(QoL_imp2_comp0$cycleno > QoL_imp2_comp0$lastcycle_no & QoL_imp2_comp0$died==1)]<-0


gee_comp0 <- glmgee(formula = QoL ~ as.factor(cycleno), data=QoL_imp2_comp0, id=pat.id ,corstr = "independence")
gee_comp0_pred.means <- data.frame(cycleno = 1:40)
gee_comp0_pred.means$mean_QoL <- predict(gee_comp0, newdata = gee_pred.means, se.fit = TRUE)[,"fit"]
gee_comp0_pred.means$se.mean_QoL <- predict(gee_comp0, newdata = gee_pred.means, se.fit = TRUE)[,"se.fit"]

deathstrategies.dat <- rbind(deathstrategies.dat, 
                             data.frame(cycleno = 1:40,
                                        QoL_mean = gee_comp0_pred.means$mean_QoL,
                                        QoL_se.mean = gee_comp0_pred.means$se.mean_QoL,
                                        strategy=rep("2. Composite strategy, QoL after death = 0", 40)))

#LINEAR MIXED MODELS -----------------------------------------
# First for hypothetical strategies
Hyp_LMM2 <- lmer(QoL ~ (1+cycleno|pat.id) + as.factor(cycleno),
                  data=QoL_imp2)

QoL_imp2$pred_HypLMM2 <- predict(Hyp_LMM2, newdata=QoL_imp2, re.form=NULL)

Hyp_LMM3 <- lmer(QoL ~ (1|pat.id) + as.factor(cycleno),
                  data=QoL_imp2)

QoL_imp2$pred_HypLMM3 <- predict(Hyp_LMM3, newdata=QoL_imp2, re.form=NULL)

AIC(Hyp_LMM2)
AIC(Hyp_LMM3)
anova(Hyp_LMM2, Hyp_LMM3)

LMM2means <- aggregate(pred_HypLMM2~cycleno, data=QoL_imp2, FUN = function(x){mean(x, na.rm=TRUE)})
LMM3means <- aggregate(pred_HypLMM3~cycleno, data=QoL_imp2, FUN = function(x){mean(x, na.rm=TRUE)})

# Obtain estimated means and standard errors
LMM2_pred.means <- data.frame(cycleno = 1:40)
LMM2_pred.means$mean_QoL <- summary(emmeans(Hyp_LMM2, ~cycleno))[,"emmean"]
LMM2_pred.means$se.mean_QoL <- summary(emmeans(Hyp_LMM2, ~cycleno))[,"SE"]

LMM3_pred.means <- data.frame(cycleno = 1:40)
LMM3_pred.means$mean_QoL <- summary(emmeans(Hyp_LMM3, ~cycleno))[,"emmean"]
LMM3_pred.means$se.mean_QoL <- summary(emmeans(Hyp_LMM3, ~cycleno))[,"SE"]

# Now use an LMM to predict only for those alive and not censored, and average over those alive
QoL_imp2_alive <- QoL_imp2[-which(QoL_imp2$cycles_till_end < 0),]
LMM2means_alive <- aggregate(pred_HypLMM2~cycleno, data=QoL_imp2_alive, FUN = function(x){mean(x, na.rm=TRUE)})

## Obtain bootstrap standard errors for the means while alive from a model object
LMM2_alive <- lmer(QoL ~ (1+cycleno|pat.id) + as.factor(cycleno),
                   data=QoL_imp2_alive)

means_WA_LMM <- function(model){
  df <- attr(model, "frame")
  df$pred <- predict(model, newdata=df, re.form=NULL)
  LMM_means_WA <- aggregate(pred~cycleno, data=df, FUN = function(x){mean(x, na.rm=TRUE)})
  LMM_means_WA_vec <- LMM_means_WA[,c("pred")]
  name_num <- c(paste0(0, 1:9), as.character(10:maxcycles))
  names(LMM_means_WA_vec) <- paste0("cycle", name_num)
  return(LMM_means_WA_vec)
}

LMM2_alive_boot <- bootstrap(model = LMM2_alive, .f=means_WA_LMM, type = "case", resample = c(TRUE, FALSE), B=1000) #~2sec / B sample
CIs_LMM_WA <- confint(LMM2_alive_boot, type="norm")
```

```
#------------PLOTS---------------
#Estimated means at all cycles
deathstrategies.dat <- rbind(deathstrategies.dat, 
                             data.frame(cycleno = 1:40,
                                        QoL_mean = LMM2_pred.means$mean_QoL,
                                        QoL_se.mean = LMM2_pred.means$se.mean_QoL,
                                        strategy=rep("3b. Hypothetical: linear mixed model, random intercept and slope", 40)),
                             data.frame(cycleno = 1:40,
                                        QoL_mean = LMM3_pred.means$mean_QoL,
                                        QoL_se.mean = LMM3_pred.means$se.mean_QoL,
                                        strategy=rep("3a. Hypothetical: linear mixed model, random intercept", 40))
                             # data.frame(cycleno = 1:40,
                             #            QoL_mean = LMM3means_alive$pred_HypLMM3,
                             #            strategy=rep("5. Linear mixed model, random intercept, avgd over those alive", 40))
                             )

deathstrategies.dat$CI_upp <- deathstrategies.dat$QoL_mean +1.96*deathstrategies.dat$QoL_se.mean
deathstrategies.dat$CI_low <- deathstrategies.dat$QoL_mean -1.96*deathstrategies.dat$QoL_se.mean

deathstrategies.dat <- rbind(deathstrategies.dat, 
                             data.frame(cycleno = 1:40,
                                        QoL_mean = LMM2means_alive$pred_HypLMM2,
                                        QoL_se.mean = NA,
                                        CI_upp = CIs_LMM_WA$upper,
                                        CI_low = CIs_LMM_WA$lower,
                                        strategy=rep("1b. While alive: linear mixed model, random icpt and slope, avgd over alive", 40))#,
                             )


death_strat <- ggplot(data = deathstrategies.dat, 
                      aes(x=cycleno, y=QoL_mean, color=strategy, fill=strategy, shape=strategy))+
  geom_point(alpha=0.9)+
  scale_shape_manual(values=c(15,5,20,3,7,8))+
  ylim(c(0,100))+
  #ggtitle("Estimated mean global QoL per treatment cycle", subtitle = "Each strategy to deal with death implies a different estimand")+
  theme_bw()+
  theme(legend.position=c(0.32, 0.23),
        legend.text=element_text(size=7.5),
        legend.background = element_rect(fill = "transparent"),
        legend.spacing.y = unit(0.1, 'cm'),
        legend.title=element_text(size=10),
        legend.key.size = unit(0.1, 'cm'))+
  guides(fill = guide_legend(byrow = TRUE),
         color = guide_legend(byrow = TRUE),
         shape = guide_legend(byrow = TRUE))+
  xlab("cycle number")+
  ylab("Estimated mean global QoL")

ggarrange(death_strat, p3, p_pd, p_td, p4, ncol = 1, heights = c(14,1,1,1,1), align = "v")

ggsave(filename = "deathstrategies.pdf", width = 8, height = 5)

# Estimated means and confidence intervals at selected cycles
death_strat_CI <- ggplot(data = deathstrategies.dat[which(deathstrategies.dat$cycleno %in% c(1,5,10,15,20,25,30,35,40)),], aes(x = cycleno, y = QoL_mean, color=strategy, fill=strategy, shape=strategy))+
  geom_point(position=position_dodge(3))+
  geom_errorbar(aes(ymin=CI_low, ymax=CI_upp), width=.5,
                position=position_dodge(3))+
  scale_shape_manual(values=c(15,5,20,3,7,8))+
  theme_classic()+
  ylab("Estimated mean global QoL with 95%CI")+
  xlab("cycle number")+
    theme(legend.position=c(0.5, 0.15),
        legend.text=element_text(size=7.5),
        legend.background = element_rect(fill = "transparent"),
        legend.spacing.y = unit(0.1, 'cm'),
        legend.title=element_text(size=10),
        legend.key.size = unit(0.1, 'cm'))+
  guides(fill = guide_legend(byrow = TRUE),
         color = guide_legend(byrow = TRUE),
         shape = guide_legend(byrow = TRUE))+
  scale_x_continuous(breaks=c(1,5,10,15,20,25,30,35,40))+
  ylim(c(15,75))
ggsave(death_strat_CI, filename="death_strat_CI.png", width = 4.5, height = 4.5)
```

### Estimating mean QoL over time under different strategies to deal with treatment discontinuation (Figure 4)

Note that some of the strategies here require the dataset QoL\_imp3,
which is the imputed dataset QoL\_imp2 with all QoL data after TD
removed.

```
# OBSERVED MEANS ---------------------------------------
TDstrategies.dat <- data.frame(cycleno = 1:40, 
                                  QoL_mean = QoL_means.dat$QoL_mean[1:40],
                                  QoL_se.mean = QoL_means.dat$QoL_se.mean[1:40],
                                  strategy=rep("0. Observed in available data", 40))


# WHILE ON TREATMENT (AND ALIVE) -------------------------

## Removing all QoL data after treatment discontinuation for while on treatment estimand
QoL_imp3 <- QoL_imp2
QoL_imp3$QoL[which(QoL_imp3$cycleno > QoL_imp3$cycle_eoTx)] <- NA

gee_TD <- glmgee(formula = QoL ~ as.factor(cycleno), data=QoL_imp3, id=pat.id ,corstr = "independence")

gee_pred.means <- data.frame(cycleno = 1:40)
gee_pred.means$mean_QoL <- predict(gee_TD, newdata = gee_pred.means, se.fit = TRUE)[,"fit"]
gee_pred.means$se.mean_QoL <- predict(gee_TD, newdata = gee_pred.means, se.fit = TRUE)[,"se.fit"]

TDstrategies.dat <- rbind(TDstrategies.dat, 
                             data.frame(cycleno = 1:40,
                                        QoL_mean = gee_pred.means$mean_QoL,
                                        QoL_se.mean = gee_pred.means$se.mean_QoL,
                                        strategy=rep("1. While on treatment (and alive)", 40)))


# Double hypothetical: for treatment discontinuation and death -------------


Hyp_LMM2 <- lmer(QoL ~ (1+cycleno|pat.id) + as.factor(cycleno),
                  data=QoL_imp3)

QoL_imp3$pred_HypLMM2 <- predict(Hyp_LMM2, newdata=QoL_imp3, re.form=NULL)

LMM2_pred.means <- data.frame(cycleno = 1:40)
LMM2_pred.means$mean_QoL <- summary(emmeans(Hyp_LMM2, ~cycleno))[,"emmean"]
LMM2_pred.means$se.mean_QoL <- summary(emmeans(Hyp_LMM2, ~cycleno))[,"SE"]


TDstrategies.dat <- rbind(TDstrategies.dat, 
                             data.frame(cycleno = 1:40,
                                        QoL_mean = LMM2_pred.means$mean_QoL,
                                        QoL_se.mean = LMM2_pred.means$se.mean_QoL,
                                        strategy=rep("2a. Hypothetical for TD and death (LMM avgd over all participants)", 40))
                          )


# Treatment policy, while alive --------------------------------------
## This is the same as the while alive from before
gee1 <- glmgee(formula = QoL ~ as.factor(cycleno), data=QoL_imp2, id=pat.id ,corstr = "independence")
gee_pred.means <- data.frame(cycleno = 1:40)
gee_pred.means$mean_QoL <- predict(gee1, newdata = gee_pred.means, se.fit = TRUE)[,"fit"]
gee_pred.means$se.mean_QoL <- predict(gee1, newdata = gee_pred.means, se.fit = TRUE)[,"se.fit"]

TDstrategies.dat <- rbind(TDstrategies.dat,
                             data.frame(cycleno = 1:40,
                                        QoL_mean = gee_pred.means$mean_QoL,
                                        QoL_se.mean = gee_pred.means$se.mean_QoL,
                                        strategy=rep("3. Treatment policy, while alive", 40)))

TDstrategies.dat$CI_upp <- TDstrategies.dat$QoL_mean +1.96*TDstrategies.dat$QoL_se.mean
TDstrategies.dat$CI_low <- TDstrategies.dat$QoL_mean -1.96*TDstrategies.dat$QoL_se.mean

#Hypothetical for treatment discontinuation, while alive ----------------
QoL_imp2_alive$pred_HypLMM2 <- predict(Hyp_LMM2, newdata=QoL_imp2_alive, re.form=NULL)
LMM2means_alive <- aggregate(pred_HypLMM2~cycleno, data=QoL_imp2_alive, FUN = function(x){mean(x, na.rm=TRUE)})

## Obtain bootstrap SEs
means_TD_WA_LMM <- function(model){
  df <- attr(model, "frame")
  pats_insamp <- unique(df$pat.id)
  df <- QoL_imp2_alive[which(QoL_imp2_alive$pat.id %in% pats_insamp),c("cycleno", "pat.id")]
  df$pred <- predict(model, newdata=df, re.form=NULL)
  LMM_means_WA <- aggregate(pred~cycleno, data=df, FUN = function(x){mean(x, na.rm=TRUE)})
  LMM_means_WA_vec <- LMM_means_WA[,c("pred")]
  name_num <- c(paste0(0, 1:9), as.character(10:maxcycles))
  names(LMM_means_WA_vec) <- paste0("cycle", name_num)
  return(LMM_means_WA_vec)
}

LMM2_TD_alive_boot <- bootstrap(model = Hyp_LMM2, .f=means_TD_WA_LMM, type = "case", resample = c(TRUE, FALSE), B=1000) #~2sec / B sample
CIs_LMM_TD_WA <- confint(LMM2_TD_alive_boot, type="norm")

plot(LMM2_TD_alive_boot)
ggsave(filename = "TD_boot.pdf", width = 6, height = 10)

TDstrategies.dat <- rbind(TDstrategies.dat,
                             data.frame(cycleno = 1:40,
                                        QoL_mean = LMM2means_alive$pred_HypLMM2,
                                        QoL_se.mean = NA,
                                        CI_upp = CIs_LMM_TD_WA$upper,
                                        CI_low = CIs_LMM_TD_WA$lower,
                                        strategy=rep("2b. Hypothetical for TD, while alive (LMM avgd over those alive)", 40))
                             )

                        
#--------PLOT----------------------------
# Estimated mean QoL at each cycle 
TD_strat <- ggplot(data = TDstrategies.dat, 
                      aes(x=cycleno, y=QoL_mean, color=strategy, fill=strategy, shape=strategy))+
  geom_point(alpha=0.9)+
  ylim(c(0,100))+
  #ggtitle("Estimated mean global QoL per treatment cycle", subtitle = "Each strategy to deal with treatment discontinuation (TD) implies a different estimand")+
  scale_shape_manual(values=c(15,7,20,18,8))+
  theme_bw()+
  theme(legend.position=c(0.4, 0.25),
        legend.text=element_text(size=7),
        legend.background = element_rect(fill = "white"),
        legend.spacing.y = unit(0.1, 'cm'),
        legend.title=element_text(size=10),
        legend.key.size = unit(0.1, 'cm'))+
  guides(fill = guide_legend(byrow = TRUE),
         color = guide_legend(byrow = TRUE))+
  xlab("cycle number")+
  ylab("Estimated mean global QoL")

ggarrange(TD_strat, p3, p_pd, p_td, p4, ncol = 1, heights = c(12,1,1,1,1), align = "v")
ggsave(filename = "TDstrategies.pdf", width = 6, height = 5)

# Estimated mean QoL and 95% confidence intervals at selected cycles
TD_strat_CI <- ggplot(data = TDstrategies.dat[which(TDstrategies.dat$cycleno %in% c(1,5,10,15,20,25,30,35,40)),], aes(x = cycleno, y = QoL_mean, color=strategy, fill=strategy, shape=strategy))+
  geom_point(position=position_dodge(3))+
  geom_errorbar(aes(ymin=CI_low, ymax=CI_upp), width=.5,
                position=position_dodge(3))+
  scale_shape_manual(values=c(15,5,20,3,7,8))+
  theme_classic()+
  ylab("Estimated mean global QoL with 95%CI")+
  xlab("cycle number")+
    theme(legend.position=c(0.5, 0.15),
        legend.text=element_text(size=7.5),
        legend.background = element_rect(fill = "transparent"),
        legend.spacing.y = unit(0.1, 'cm'),
        legend.title=element_text(size=10),
        legend.key.size = unit(0.1, 'cm'))+
  guides(fill = guide_legend(byrow = TRUE),
         color = guide_legend(byrow = TRUE),
         shape = guide_legend(byrow = TRUE))+
  scale_x_continuous(breaks=c(1,5,10,15,20,25,30,35,40))+
  ylim(c(15,75))
ggsave(TD_strat_CI, filename="TD_strat_CI.png", width = 4.5, height = 4.5)
```

### Estimating mean QoL over time under different strategies to deal with disease progression (Figure 5)

Note that here, the dataset QoL\_imp3 is redefined as the imputed
dataset QoL\_imp2 with all QoL data after PD removed.

```
QoL_imp3 <- QoL_imp2

#Removing all data after PD for our illustration
QoL_imp3$QoL[which(QoL_imp3$cycleno > QoL_imp3$cycle_PD)] <- NA

# OBSERVED MEANS --------------------------------------------------
PDstrategies.dat <- data.frame(cycleno = 1:40, 
                                  QoL_mean = QoL_means.dat$QoL_mean[1:40],
                                  QoL_se.mean = QoL_means.dat$QoL_se.mean[1:40],
                                  strategy=rep("0. Observed in available data", 40))

# While no PD (and alive)-----------------------------------------
gee_PD <- glmgee(formula = QoL ~ as.factor(cycleno), data=QoL_imp3, id=pat.id ,corstr = "independence")

gee_pred.means <- data.frame(cycleno = 1:40)
gee_pred.means$mean_QoL <- predict(gee_PD, newdata = gee_pred.means, se.fit = TRUE)[,"fit"]
gee_pred.means$se.mean_QoL <- predict(gee_PD, newdata = gee_pred.means, se.fit = TRUE)[,"se.fit"]

PDstrategies.dat <- rbind(PDstrategies.dat, 
                             data.frame(cycleno = 1:40,
                                        QoL_mean = gee_pred.means$mean_QoL,
                                        QoL_se.mean = gee_pred.means$se.mean_QoL,
                                        strategy=rep("1. While without disease progression (and alive)", 40)))

# Double hypothetical: for PD and death---------------------------


Hyp_LMM2 <- lmer(QoL ~ (1+cycleno|pat.id) + as.factor(cycleno),
                  data=QoL_imp3)

QoL_imp3$pred_HypLMM2 <- predict(Hyp_LMM2, newdata=QoL_imp3, re.form=NULL)


LMM2_pred.means <- data.frame(cycleno = 1:40)
LMM2_pred.means$mean_QoL <- summary(emmeans(Hyp_LMM2, ~cycleno))[,"emmean"]
LMM2_pred.means$se.mean_QoL <- summary(emmeans(Hyp_LMM2, ~cycleno))[,"SE"]


PDstrategies.dat <- rbind(PDstrategies.dat, 
                             data.frame(cycleno = 1:40,
                                        QoL_mean = LMM2_pred.means$mean_QoL,
                                        QoL_se.mean = LMM2_pred.means$se.mean_QoL,
                                        strategy=rep("2a. Hypothetical for PD and death (LMM avgd over all participants)", 40))#,
                          )


# Treatment policy, while alive---------------------------------------
#This is the while alive from above
gee1 <- glmgee(formula = QoL ~ as.factor(cycleno), data=QoL_imp2, id=pat.id ,corstr = "independence")
gee_pred.means <- data.frame(cycleno = 1:40)
gee_pred.means$mean_QoL <- predict(gee1, newdata = gee_pred.means, se.fit = TRUE)[,"fit"]
gee_pred.means$se.mean_QoL <- predict(gee1, newdata = gee_pred.means, se.fit = TRUE)[,"se.fit"]

PDstrategies.dat <- rbind(PDstrategies.dat,
                             data.frame(cycleno = 1:40,
                                        QoL_mean = gee_pred.means$mean_QoL,
                                        QoL_se.mean = gee_pred.means$se.mean_QoL,
                                        strategy=rep("3. Treatment policy, while alive", 40)))

#add CIs

PDstrategies.dat$CI_upp <- PDstrategies.dat$QoL_mean +1.96*PDstrategies.dat$QoL_se.mean
PDstrategies.dat$CI_low <- PDstrategies.dat$QoL_mean -1.96*PDstrategies.dat$QoL_se.mean


#Hypothetical for PD, while alive ----------------------------------------
QoL_imp3_alive <- QoL_imp3[-which(QoL_imp3$cycles_till_end < 0),]
LMM2means_alive <- aggregate(pred_HypLMM2~cycleno, data=QoL_imp3_alive, FUN = function(x){mean(x, na.rm=TRUE)})

means_PD_WA_LMM <- function(model){
  df <- attr(model, "frame")
  pats_insamp <- unique(df$pat.id)
  df <- QoL_imp2_alive[which(QoL_imp2_alive$pat.id %in% pats_insamp),c("cycleno", "pat.id")]
  df$pred <- predict(model, newdata=df, re.form=NULL)
  LMM_means_WA <- aggregate(pred~cycleno, data=df, FUN = function(x){mean(x, na.rm=TRUE)})
  LMM_means_WA_vec <- LMM_means_WA[,c("pred")]
  name_num <- c(paste0(0, 1:9), as.character(10:maxcycles))
  names(LMM_means_WA_vec) <- paste0("cycle", name_num)
  return(LMM_means_WA_vec)
}

require(lmeresampler)
LMM2_PD_alive_boot <- bootstrap(model = Hyp_LMM2, .f=means_PD_WA_LMM, type = "case", resample = c(TRUE, FALSE), B=1000) #~2sec / B sample
CIs_LMM_PD_WA <- confint(LMM2_PD_alive_boot, type="norm")

plot(LMM2_PD_alive_boot)
ggsave(filename = "PD_boot.pdf", width = 6, height = 10)


PDstrategies.dat <- rbind(PDstrategies.dat,
                             data.frame(cycleno = 1:40,
                                        QoL_mean = LMM2means_alive$pred_HypLMM2,
                                        QoL_se.mean = NA,
                                        CI_upp = CIs_LMM_PD_WA$upper,
                                        CI_low = CIs_LMM_PD_WA$lower,
                                        strategy=rep("2b. Hypothetical for PD, while alive (LMM avgd over those alive)", 40))
                             )


#---------------PLOTS------------------------------
# Estimated mean QoL at each cycle
PD_strat <- ggplot(data = PDstrategies.dat, 
                      aes(x=cycleno, y=QoL_mean, color=strategy, fill=strategy, shape=strategy))+
  geom_point(alpha=0.8)+
  ylim(c(0,100))+
  #ggtitle("Estimated mean global QoL per treatment cycle", subtitle = "Each strategy to deal with disease progression (PD) implies a different estimand")+
  scale_shape_manual(values=c(0,7,20,18,8))+
  theme_bw()+
  theme(legend.position=c(0.4, 0.25),
        legend.text=element_text(size=7),
        legend.background = element_rect(fill = "white"),
        legend.spacing.y = unit(0.1, 'cm'),
        legend.title=element_text(size=10),
        legend.key.size = unit(0.1, 'cm'))+
  guides(fill = guide_legend(byrow = TRUE),
         color = guide_legend(byrow = TRUE))+
  xlab("cycle number")+
  ylab("Estimated mean global QoL")

ggarrange(PD_strat, p3, p_pd, p_td, p4, ncol = 1, heights = c(12,1,1,1,1), align = "v")
ggsave(filename = "PDstrategies.pdf", width = 6, height = 5)

# Estimated means and 95% confidence intervals at selected cycles
PD_strat_CI <- ggplot(data = PDstrategies.dat[which(PDstrategies.dat$cycleno %in% c(1,5,10,15,20,25,30,35,40)),], aes(x = cycleno, y = QoL_mean, color=strategy, fill=strategy, shape=strategy))+
  geom_point(position=position_dodge(3))+
  geom_errorbar(aes(ymin=CI_low, ymax=CI_upp), width=.5,
                position=position_dodge(3))+
  scale_shape_manual(values=c(15,5,20,3,7,8))+
  theme_classic()+
  ylab("Estimated mean global QoL with 95%CI")+
  xlab("cycle number")+
    theme(legend.position=c(0.5, 0.15),
        legend.text=element_text(size=7.5),
        legend.background = element_rect(fill = "transparent"),
        legend.spacing.y = unit(0.1, 'cm'),
        legend.title=element_text(size=10),
        legend.key.size = unit(0.1, 'cm'))+
  guides(fill = guide_legend(byrow = TRUE),
         color = guide_legend(byrow = TRUE),
         shape = guide_legend(byrow = TRUE))+
  scale_x_continuous(breaks=c(1,5,10,15,20,25,30,35,40))+
  ylim(c(15,75))
ggsave(PD_strat_CI, filename="PD_strat_CI.png", width = 4.5, height = 4.5)
```
